# Sex-based metabolic and microbiota differences in roots and rhizosphere soils of dioecious papaya (*Carica papaya* L.)

**DOI:** 10.3389/fpls.2022.991114

**Published:** 2022-10-13

**Authors:** Yongmei Zhou, Ziqin Pang, Zhaonian Yuan, Nyumah Fallah, Haifeng Jia, Ray Ming

**Affiliations:** ^1^ FAFU and UIUC Joint Center for Genomics and Biotechnology, Fujian Provincial Key Laboratory of Haixia Applied Plant Systems Biology, Fujian Agriculture and Forestry University, Fuzhou, China; ^2^ Key Laboratory of Sugarcane Biology and Genetic Breeding, Ministry of Agriculture, Fujian Agriculture and Forestry University, Fuzhou, China; ^3^ Department of Plant Biology, University of Illinois at Urbana-Champaign, Urbana, IL, United States

**Keywords:** root, rhizosphere soil, metabolome, microbiome, dimorphism, papaya

## Abstract

Dioecious plant species have a high genetic variation that is important for coping with or adapting to environmental stress through natural selection. Intensive studies have reported dimorphism morphism in morphology, physiology, as well as biotic and abiotic stress responses in dioecious plants. Here, we demonstrated the dimorphism of metabolic profile and the preference of some microorganisms in the roots and rhizosphere soils of male and female papaya. The metabolic composition of roots were significantly different between the males and females. Some sex hormones occurred in the differential metabolites in roots and rhizosphere soils. For example, testosterone was up-regulated in male papaya roots and rhizosphere soils, whereas norgestrel was up-regulated in the female papaya roots, indicating a possible balance in papaya roots to control the sexual differentiation. Plant hormones such as BRs, JAs, SA and GAs were also detected among the differential metabolites in the roots and rhizosphere soils of dioecious papaya. In addition, some metabolites that have medicinal values, such as ecliptasaponin A, crocin, berberine and sapindoside A were also expressed differentially between the two sexes. Numerous differential metabolites from the papaya roots were secreted in the soil, resulting in the differences in microbial community structure in the roots and rhizosphere soils. Some nitrogen-fixing bacteria such as *Allorhizobium*-*Neorhizobium*-*Pararhizobium*-*Rhizobium*, *Brevundimonas* and *Microvirga* were enriched in the male papaya roots or rhizosphere soils. While *Candidatus Solibacter* and *Tumebacillus*, which utilize organic matters, were enriched in the roots or rhizosphere soils of the female papaya. Some differences in the fungi abundance were also observed in both male and female papaya roots. These findings uncovered the effect of sex types on the metabolic and microbiota differences in roots and rhizosphere soils in papaya and will lead to investigations of underlining genomic and molecular mechanisms.

## Introduction

Most angiosperm species are hermaphroditic, with individuals having both male and female sexual organs within the same flower. However, approximately 6% of angiosperm species are dioecious (Ricklefs, 1995; Renner, 2014), which are to have evolved from ancestral hermaphroditism (Endress and Doyle, 2015; Yakimowski and Barrett, 2016). Dioecious plants play an important role in protecting species diversity and maintaining ecosystem stability. Research has shown that males and females typically have dimorphism morphism in morphology, physiology, defence and patterns of allocation to life history (Liu et al., 2021b). Compared with female plants, male plants tend to be more tolerant to environmental stresses, such as drought, salinity, heavy metals, and nutrient deficiency (Melnikova et al., 2017; Xia et al., 2020).

Papaya (*Carica papaya*.L) is an important fruit crop, known for its nutritional benefits and medicinal applications. It is cultivated in tropical and sub-tropical regions, belonging to the genus *Carica* of the family Caricaceae. All species in *Carica* are dioecious, expect one monoecious species (Decraene and Smets, 1999). Papaya is one of the two species in Caricaceae that has three sex types, including female, male and bisexual. Since pear-shaped papayas produced by hermaphrodites are more popular in the market, while the cultivated type is mostly gynodioecious. The wild papayas are dioecious with a strong dimorphism and is an ideal model to study its different responses to sexual identity. However, a number of studies have demonstrated that there are no apparent differences in the morphology between the male and female plants during the vegetative phase. Only after flowering, the males and females show many floral morphological differences. For instance, male inflorescences bear more flowers than female ones, and flowers of female papaya are larger than males in size (Decraene and Smets, 1999).

The sex in wild papaya is controlled by XY chromosomes, XX for females, and XY for males (Ming et al., 2008). Previous studies have suggested that sex differentiation in papaya may be regulated by transcription, epigenetic DNA methylation and phytohormone (Zhou et al., 2020; Liu et al., 2021a; Zhou et al., 2022). However, a vast majority of these studies have focused on the aboveground parts of dioecious plants such as flower buds, and shoot meristems, while few reports focused on the belowground parts. For example, the response of metabolic profiles and microbial communities in roots and rhizosphere soils of dioecious papaya to sex identity is still unclear.

Research on metabolites has been a hotspot in biological research because biochemical phenotype largely reflects the development stages of plants and their interactions with the environment (Carreno-Quintero et al., 2013). It is estimated that there are approximately 200, 000 structurally distinct compounds (Dixon and Strack, 2003). The phytochemical components help us effectively understand the biological processes of plants and their mechanisms. Moreover, the microbiome is strongly associated with host plant by forming a dynamically balanced ecosystem in the process of co-evolution (Compant et al., 2019; Canto et al., 2020). The microorganisms rely on the suitable habitat and nutrients provided by plants. On the other hand, microorganisms promote plant growth, nutrient uptake, photosynthesis, and stress resilience (Yu et al., 2019; Xia et al., 2021). In this study, we demonstrate the sexual differences in metabolomes and microbial communities, which can help us better understand their sex specificity of papaya.

## Materials and methods

### Sampling and preparation of roots and rhizosphere soils

Papaya variety Zhonghuang seeds were planted into the soil at a greenhouse under 30°C, with 50% relative humidity at 16:8 (light: dark) photoperiod. After 3 months, the plants were planted in field at the experimental base of Fujian Agriculture and Forestry University (25°13′15″N, 117°41′55″E), Wufeng Town, Yongchun County, Quanzhou City, Fujian Province, China. About 2 months later, the papaya began to flower, the roots and rhizosphere soils of the male and female plants were collected and stored Whirl-Pak^®^ bags. Each sample consisted of three replicates. The samples were stored in an ice box and transported immediately to the laboratory. The roots were washed using sterilized ddH2O, and then surface-sterilized with 100% ethanol for 1 min, 2.5% fresh bleach for 30 min and 100% ethanol for 1 min before storing at -80°C. The rhizosphere soils was detached from the roots of papaya. The soil of each sample was mixed, and visible roots and stones were removed before storing at -80°C (Pang et al., 2022a).

### Metabolites extraction and LC-MS/MS analysis

LC-MS/MS analyses were performed to identify exometabolites across samples using a UHPLC system (1290, Agilent Technologies) with a UPLC BEH Amide column (1.7μm 2.1*100mm, Waters) coupled to TripleTOF 5600 (Q-TOF, AB Sciex). Then 100μg of each sample was extracted with 300μL of methanol, 20μL internal standard substances with vortex was added for 30s, and was subsequently treated with ultrasound for 10min (incubated in ice water) and incubation for 1h at -20C to precipitate proteins. After centrifugation at 13,000rpm for 15 minutes at 4°C, the supernatant was transferred into a fresh 2mL LC/MS glass vial. 20μL supernatant from each sample was pooled and 200μL mixed supernatant was taken for the UHPLC-QTOF-MS analysis.

The mobile phase consisted of 25mM NH_4_OAc and 25mM NH_4_OH in water(pH=9.75)(A) and acetonitrile (B) was conducted using elution gradient as follows: 0 min, 95% B; 7min, 65% B; 9 min, 40% B; 9.1 min, 95% B; 12 min, 95% B, which was delivered at 0.5mL min^-1^. The injection volume was 3μL. The Triple TOF mass spectrometer was used for its ability to acquire MS/MS spectra on an information-dependent basis (IDA) during an LC/MS experiment. In this mode, the acquisition software (Analyst TF 1.7, AB Sciex) continuously evaluates the full scan survey MS data. In each cycle, 12 precursor ions whose intensity greater than 100 were chosen for fragmentation at collision energy (CE) of 30 V. ESI source conditions were set as follows: Ion source gas 1 as 60 Psi, Ion source gas 2 as 60 Psi, Curtain gas as 35 Psi, source temperature 650C, Ion Spray Voltage Floating (ISVF) 5000 V or -4000 V in positive or negative modes, respectively.

### Data annotation

The raw data files were converted to the mzXML format using ProteoWizard, and processed by R package XCMS (version 3.2). The preprocessing results generated a data matrix that consisted of the retention time (RT), massto-charge ratio (m/z) values, and peak intensity. R package CAMERA was used for peak annotation after XCMS data processing. In-house MS2 database was applied in metabolites identification.

### DNA extraction, and library construction for Illumina MiSeq sequencing

Total genomic DNA was extracted using the Fast DNATM Spin Kit (MP Biomedicals, LLC, Santa Ana, CA, United States). The DNA purity and quantity were determined by the NanoDrop 2000 spectrophotometer (Thermo Fisher) and agarose gel. Using the genomic DNA as a template, the hypervariable V3-V4 regions of the 16S rRNA gene were amplified by PCR with the primers 341F (5′-CCTACGGGNBGCASCAG-3′) and 785R (5′-CCTACGGGNBGCASCAG-3′) (Fadeev et al., 2021). The fungal ITS1 regions of ITS were amplified by PCR with the primers ITS5-1737F (5′- GGAAGTAAAAGTCGTAACAAGG-3′) and ITS2-2043R (5′- GCTGCGTTCTTCATCGATGC-3′) (Huang et al., 2016). The PCR amplification products were collected from a 2% agarose gel and purified by Vazyme VAHTSTM DNA Clean Beads. The sequencing libraries were established using TruSeq Nano DNA LT Library Prep Kit (Illumina, SD, USA) and then sequenced on an Illumina MiSeq platform (Biomarker Technologies Corporation, Beijing, China).

### Data preprocessing and annotation

The Illumina paired-end raw data were quality filtered using Trimmomatic (Bolger et al., 2014), and then the primer sequences were identified and removed using Cutadapt (Martin, 2011), followed by paired-end pairing using USEARCH (Edgar, 2013). Afterwards, the chimera readings were detected and removed by using the UCHIME (Edgar et al., 2011) to obtain high-quality sequences for subsequent analysis (Edgar et al., 2011). Sequences with 97% similarity were clustered at the sequence level using USEARCH with a default threshold of 0.005% of all sequences to filter operational taxonomic units (OTUs) (Edgar, 2013). To annotate the taxonomic information for the sequences, Ribosomal Database Project (RDP) classifier was used to annotate the species of all representative reads with a confidence threshold of 70% according to the Silva database (version 138) (Wang et al., 2007).

### Statistical analysis

Shannon index and richness index (ACE) estimator were used to analyze the alpha diversity by using phyloseq package (McMurdie and Holmes, 2013). For beta diversity analysis, Principal Component Analysis (PCoA) was performed using Bray-Curtis algorithm of Quantitative Insights into Microbial Ecology (QIIME) and R software (version 4.1.3) to compare the similarity of species diversity in the different samples (Suchodolski et al., 2012). We later conducted Permutational Multivariate Analysis of Variance (PERMANOVA) and paired PERMANOVA using vegan package at 999 permutations and α = 0.05 to test metabolites dissimilarities between sexes. The depleted or enriched metabolites and microbial genera in each sex were determined on the criteria having an P value < 0.05 and fold change value >2. Manhattan plot and volcano plot were employed using the R language to illustrate the differential metabolites and differential microbes of the roots and rhizosphere soils between the two sexes. The correlations between differential metabolites and differential microbes were visualized using a correlation matrix by computing all potential pairwise Spearman’s rank using Cytoscape version 3.6.1 (Shannon et al., 2003). We used the peak area intensity of metabolites and the relative abundance of microorganisms Pearson correlation analysis. The co-occurrence network analysis was based on Pearson correlation (p) was >0.9 and the P-value was < 0.05.

## Results

### Metabolic composition and beta diversity in roots and rhizosphere soils between sexes

**Figure 1:**
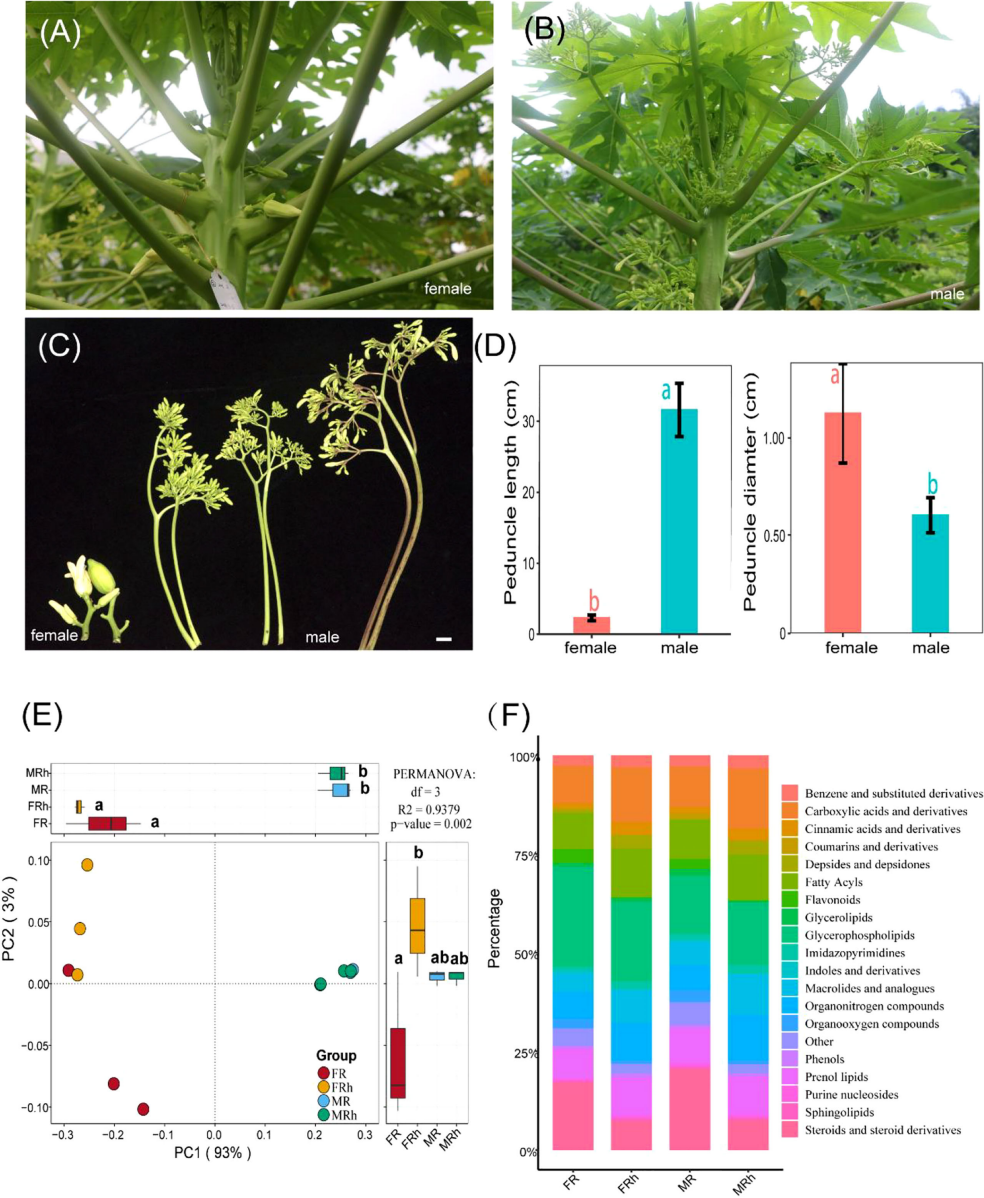
**(A-D)** Male papaya bears a longer but less thick peduncle with numerous flowers. Bar = 2cm. **(E)** Principal coordinates analysis (PCoA) plot based on unweighted Unifrac distance (β-diversity) on Bray-Curtis distance showing the separation of the samples according to metabolite profile of the roots and rhizosphere soils of the female and male papaya. PC1 and PC2 together explained 96% of the information in metabolic composition. The differential analysis was performed on PC1 and PC2 to reveal the differences in metabolic composition. Different letters represented significant differences (P < 0.05). Additionally, there were significant differences between every two groups in Permanova analysis (P < 0.01). Each point corresponded to a sample. **(F)** Relative abundance of Top 19 metabolic taxonomy in the entire sample. FR, female papaya roots; FRh, female papaya rhizosphere soils; MR, male papaya roots; MRh, male papaya rhizosphere soils.

We investigated the morphological dimorphism of inflorescences in dioecious papaya. Compared with the females, male papaya showed longer peduncles,beraing numerous flowers at different stages (**Figures 1A, B**). This morphology ensures male papaya take full advantage of space and produce pollen grains continually. While female papaya showed short but thicker peduncle (**Figures 1C, D**).

LC-MS/MS non-targeted analyses were conducted to identify metabolites in roots and rhizosphere soils of dioecious papaya based on The Human Metabolome Database (HMDB). A total of 2602 metabolites were detected in the entire sample. The metabolites were classified into 80 taxonomic categories, including fatty acyls, coumarins and derivatives, followed by indoles and derivatives and phenols.

The PCoA analysis showed that there were significant difference in root and rhizosphere soil metabolites between females and males (P < 0.01 in Permanova analysis). The first (PC1) and second (PC2) principal component explained 77% and 8% of the variance in the metabolic composition, respectively. The metabolite compositions of roots and rhizosphere soils were distinctly separated along the first major axis, indicating that the differences in root and rhizosphere soil of the males and females were more significant (**Figure 1E**). The inter-group difference analysis of PC2 showed that the root metabolic composition of male papaya was significantly different to female papaya.

In addition, the differences between the roots and rhizosphere soils of the male papaya were smaller than those observed in the female papaya. Moreover, we also identified the top twenty relative abundance of metabolic taxonomy, including glycerophospholipids (14.8%-25.3%), steroids and steroid derivatives (7.4%-20.5%), carboxylic acids and derivatives (9.6%-15.3%), fatty acyls (9.1%-12.5%), prenol lipids (7.6%-9.9%), organonitrogen compounds (6.3%-11.6%), macrolides and analogues (4.6%-10.3%), flavonoids (0.0%-3.5%), and phenols (0.8%-1.1%) (**Figure 1F**). We performed multiple difference comparison analysis to test the significant difference of these taxa in different compartments under both groups. Compared with the roots and rhizosphere soils of the males plant, the relative abundance of steroids and steroid derivatives, organooxygen compounds, flavonoids and indoles and derivatives of the female roots and rhizosphere soils were significantly higher (P < 0.05), while the abundance of Cinnamic acids and derivatives showed an opposite trend (**Table S1**).

### Differential aboundance analysis of metabolites in roots and rhizosphere soils between females and males

**Figure 2:**
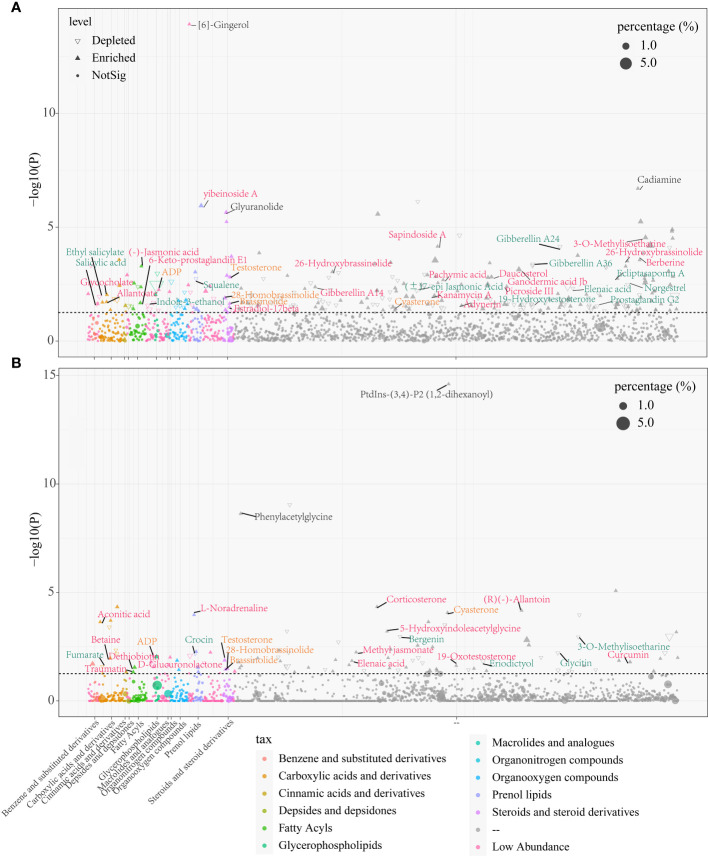
Manhattan plot showing differences in metabolites between the male and female papaya in the roots and rhizosphere soils, **(A)** papaya roots; **(B)** papaya rhizosphere soils. Each point represented a metabolite, and its size depicted the relative abundance of the metabolite, and the colors of the point denoted the metabolite categories. The P threshold of the dotted line was about 1.30 [-log10 (0.05)], and metabolites above the dashed line represents the metabolites with significant differences between males and females. The upward triangles showed that the metabolites of the males were significantly higher than those of the females, while the downward triangles of the males represented significantly lower metabolites than those of the females. Each dot signified no difference in substance between the males and females. Metabolites in black font were the ones with the highest change fold. Red font indicated metabolites that were significantly enriched in the males. Orange font indicated metabolites that were significantly increased in both roots and rhizosphere soils of the male, while green font indicated metabolites that were significantly lower in the males. “–” in taxonomy indicated metabolites which was not currently classified in the HMDB database, accounting for more than 75% of the total amount of substances detected.

To reveal the differences in metabolites of both sexes in the rhizosphere soils and roots, we adopted DESeq2 analysis and later visualized using Manhattan plot. The results showed that there were numerous differential metabolites in papaya roots and rhizosphere soils of both female and male papaya, with the female accounting for 400 metabolites and 173 metabolites for the male. Among them, the abundance of gibberellin A24, gibberellin A36, indole-3-ethanol, squalene, ecliptasaponin A, 3-O-Methylisoetharine and norgestrel were found significantly higher in the female papaya roots than in male papaya. However, the abundance of 26-Hydroxybrassinolide, (-)-Jasmonic acid, glycocholate, gibberellin A14, picroside III, sapindoside A, 6-Keto-prostaglandin E1 and 3-O-Methylisoetharine showed the opposite trend (**Figure 2A**; **Table S2A**). The abundance of metabolites such as eriodictyol and fumarate in the female papaya rhizosphere soils was significantly increased, while testosterone, aconitic acid, 5-Hydroxyindoleacetylglycine, betaine, 19-Oxotestosterone and others were significantly increased in the male papaya rhizosphere soils. Some metabolites such as testosterone, ADP, 28-Homobrassinolide and brassinolide were significantly increased in both roots and rhizosphere soils of male papaya (**Figure 2B**; **Table S2B**).

**Figure 3:**
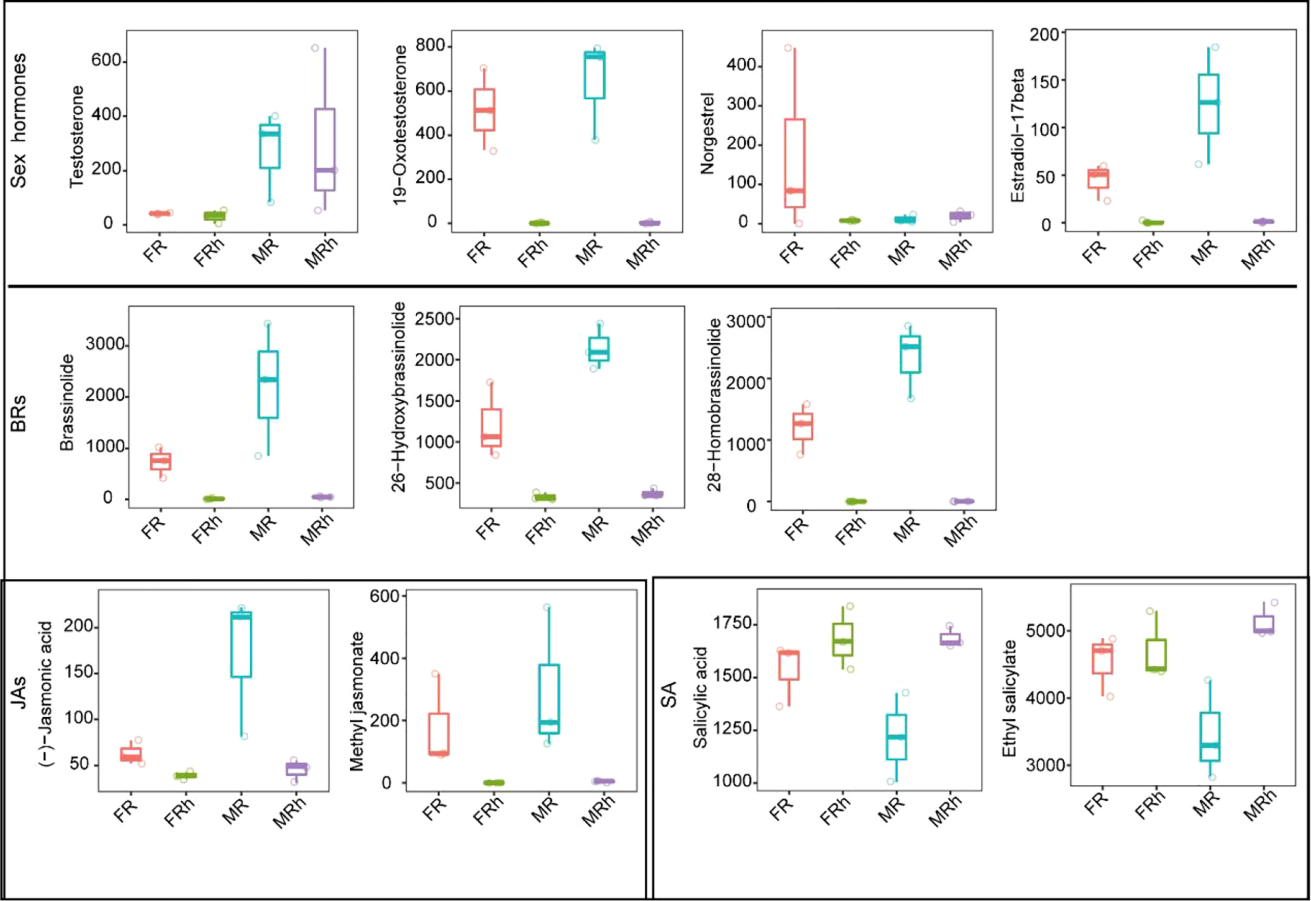
The compare analysis of hormone relative abundance in the roots and rhizosphere soils of papaya.

The sex hormones occurred in the differential metabolites in roots and rhizosphere soils. For example, testosterone was up-regulated in male papaya roots and rhizosphere soils, while norgestrel was up-regulated in the female papaya roots (**Figure 3**). Plant hormones such as BRs, JAs, SA and GAs were also differentially expressed in the roots and rhizosphere soils of dioecious papaya (**Figure 3**).

### Overview of microbial communities in roots and rhizosphere soils

For 16S rRNA sequencing data, a total of 959,962 pairs of reads were obtained from 12 samples, and 956,415 clean reads were generated after paired-end reads quality control and splicing. Each sample produced at least 76,196 clean reads, with an average of 79,701 clean reads. The average GC content of 16S bacterial rRNA was 55.48%, and the bases with a base quality value greater than or equal to 30 accounted for 96.67% of the total bases. We obtained 1,806 OTUs at 97% identity from the 12 samples, with the number ranging from 824 to 1,767 per sample (**Table S3A**). The coverage for the observed OTUs was 99.81 ± 0.01% (mean ± sem) and the rarefaction curves showed clear asymptotes (**Figure S1A**), which demonstrated a near-complete sampling of the community.

To identify fungal species, the ITS1 region of the ITS was amplified and sequenced using the DNA from the roots and rhizosphere soils of papaya. A total of 511,385 pairs of reads were obtained from the 12 samples, and a total of 508,494 clean reads were filtered for splicing. Each sample generated at least 26,121 clean reads, with an average of 42,374 clean reads.

The average GC content of ITS1 was 45.76%, with bases containing a quality value greater than or equal to 30, accounting for 99.14% of the total number of bases. We obtained 1,021 OTUs at 97% identity from the entire sample, with the number of OTUs ranging from 268 to 638 per sample (**Table S3B**). The coverage for the observed OTUs was 99.84 ± 0.04% (mean ± sem) and the rarefaction curves showed clear asymptotes (**Figure S1B**), which demonstrated a near-complete sampling of the community.

**Figure 4:**
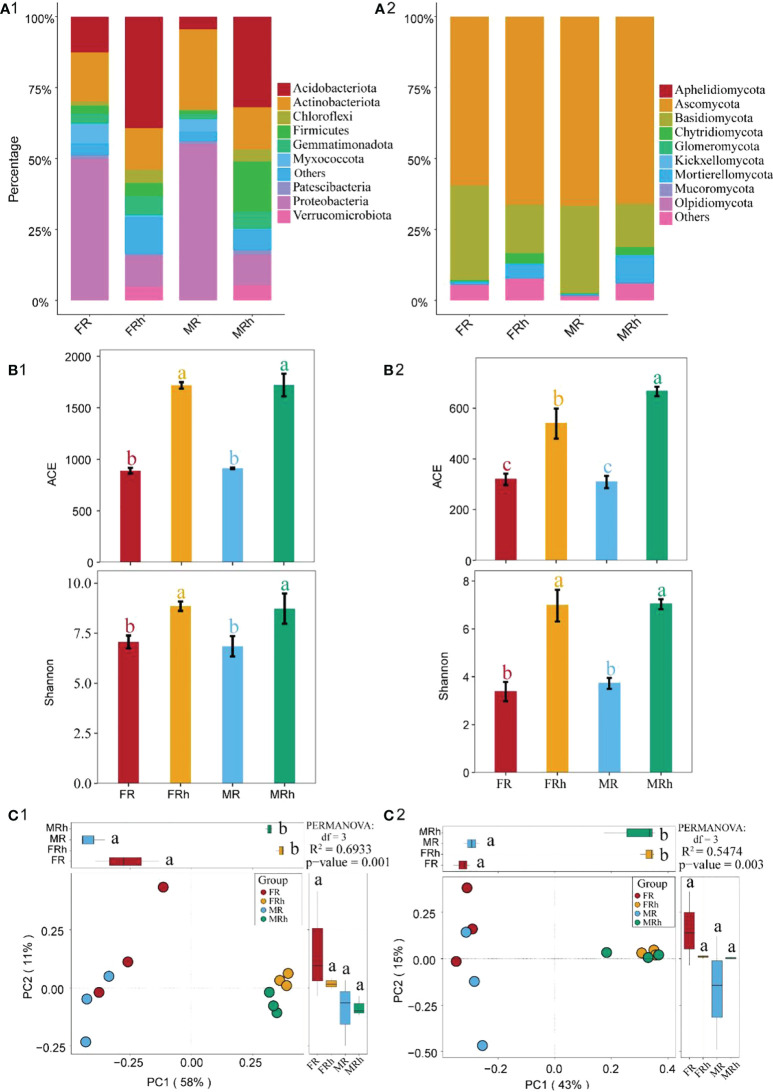
Taxonomic composition of the bacteria **(A1)** and fungi **(A2)** in the roots and rhizosphere soils of the female and male papaya. Alpha diversity analysis showed the richness index (ACE index) and diversity index (Shannon index) of bacteria **(B1)** and fungi **(B2)** in the roots and rhizosphere soils between the male and female papaya. The different letters meant a significant difference (p< 0.05). PCoA analysis demonstrating bacteria **(C1)** and fungal **(C2)** diversity in the roots and rhizosphere soils between the female and male papaya. The differential analysis was performed on PC1 and PC2 to reveal the differences in microbial composition. Different letters represented significant differences (P < 0.05). Permanova analysis revealed significant differences between every two sample groups (P < 0.01).

Numerous bacteria and fungi were detected in papaya roots and rhizosphere soils by16S rRNA/ITS sequencing. Proteobacteria (10.8%-55.1%), Acidobacteriota (4.4%-39.3%) and Actinobacteriota (14.7%-28.2%) were dominant bacteria identified, followed by Firmicutes (1.2%-17.6%) and Gemmatimonadota (1.8%-6.9%) (**Figure 4A1**). Notably, the relative abundances of Proteobacteria and Myxococcota in papaya roots were significantly increased (p < 0.05) compared with rhizosphere soils, while the opposite results were found for Acidobacteriota (**Table S4**). On the other hand, Ascomycota (59.5%-66.7%) and Basidiomycota (15.2%-33.3%) were the dominant fungi in papaya root and rhizosphere soils, followed by Chytridiomycota (0.2%-3.5%) (**Figure 4A2**). The relative abundance of Chytridiomycota in papaya roots was significantly (p < 0.05) lower than in papaya rhizosphere soils (**Table S4**).

### Alpha and beta diversity for bacteria and fungi between sexes

We further investigated the bacterial and fungal richness (ACE) and diversity (Shannon) indices in roots and rhizosphere soils of the female and male papaya. The results showed that compared with the rhizosphere soils, the ACE and Shannon index of bacteria and fungi in the roots of both female and male papaya were significantly decreased (p< 0.05), while the difference between the two sexes was not significant (**Figures 4B1, B2**).

The OTUs of 16S rRNA/ITS sequencing were used for PCoA analysis which illustrated the overall similarity of the bacterial and fungal community composition between samples. PCoA plot based on unweighted Unifrac distance (β-diversity) on Bray-Curtis distance showing the separation of the samples according to the composition of bacteria and fungi in roots and rhizosphere soils between the female and male papaya. PC1 and PC2 explained 58% and 11% of the total variance in bacterial community composition, respectively (**Figure 4C1**). PC1 and PC2 also explained 43% and 15% of the total variance in fungal community composition, respectively (**Figure 4C2**). The results showed that the microbial compositions in the roots and rhizosphere soils were significantly separated along the first axis, suggesting that the microbial differences in soil and roots were more pronounced (**Figures 4C1, C2**).

### Differential abundance analysis of bacterial and fungal genera

**Figure 5:**
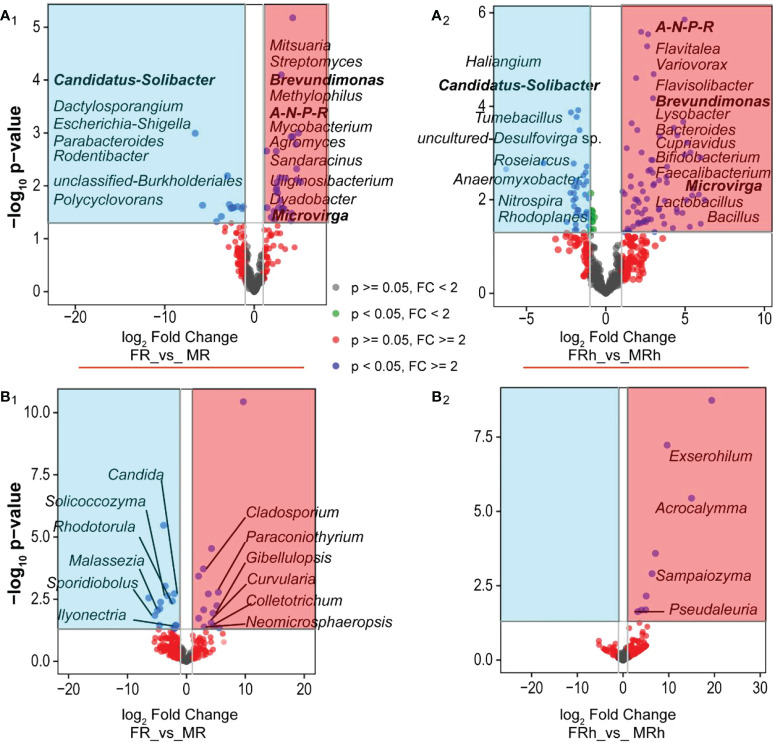
Volcano plots depicting the differential abundance of microbial communities (genus level) in the roots and rhizosphere soils of the female and male. **(A)**, bacteria; **(B)**, fungi; 1, roots; 2, rhizosphere soils. *A-N-P-R* is the abbreviation of *Allorhizobium-Neorhizobium-Pararhizobium-Rhizobium*. The spots in the blue background represented significantly up-regulated in the females, while the spots in the red background were significantly up-regulated in the males. The bold black font indicated the significantly changed microorganisms in both male and female papaya roots and rhizosphere soils.

To identify the differential abundance of bacteria and fungi of the two sex types in roots and rhizosphere soils, DESeq2 was employed and visualized using volcano plot. The results showed that the sex-differentiated bacteria in roots and rhizosphere soils were 40 and 119 genera (**Figures 5A1, A2**, **Tables S5A, B**), respectively, while the differential fungi were 28 and 9 genera (**Figures 5B1, B2**, **Tables S5C, D**), respectively. In the roots, the abundance of bacteria such as *Candidatus-Solibacter*, *Parabacteroides*, *Dactylosporangium*, *Escherichia_Shigella*, *Rodentibacter*, *unclassified-Burkholderiales* and *Polycyclovorans* were significantly higher than the female papaya’s, whereas the abundance of *Allorhizobium-Neorhizobium-Pararhizobium-Rhizobium*, *Brevundimonas*, *Microvirga*, *Streptomyces*, *Agromyces*, *Mitsuaria*, *Uliginosibacterium*, *Mycobacterium, Sandaracinus*, *Dyadobacter*, and *Methylophilus* were significantly lower than males (**Figure 5A1**). The female papaya rhizosphere soils was significantly enriched with *Tumebacillus*, *Roseiarcus*, *Haliangium*, *Anaeromyxobacter*, *Nitrospira*, *uncultured-Desulfovirga* sp., and *Rhodoplanes*. While male papaya rhizosphere soils was enriched the bacterial genera including *Brevundimonas*, *Bifidobacterium*, *Flavitalea*, *Flavisolibacter*, *Lactobacillus*, *Microvirga*, *Cupriavidus, Faecalibacterium*, *Lysobacte*, *Variovorax*, *Bacteroides* and *Bacillus* than male (**Figure 5A2**). For fungi, the abundance of *Candida*, *Solicoccozyma*, *Rhodotorula*, etc. were significantly higher in the female papaya roots than in the males. Conversely, the abundance of, *Paraconiothyrium*, *Gibellulopsis* and *Curvularia* were significantly lower in the male papaya roots compared with the male papaya roots (**Figure 5B1**). Moreover, the abundance of *Exserohilum*, *Acrocalymma*, *Sampaiozyma* and *Pseudaleuria* in the rhizosphere soils of the female papaya was significantly lower than that of the males (**Figure 5B2**).

### Relationships between microbial and metabolomic profiles

**Figure 6:**
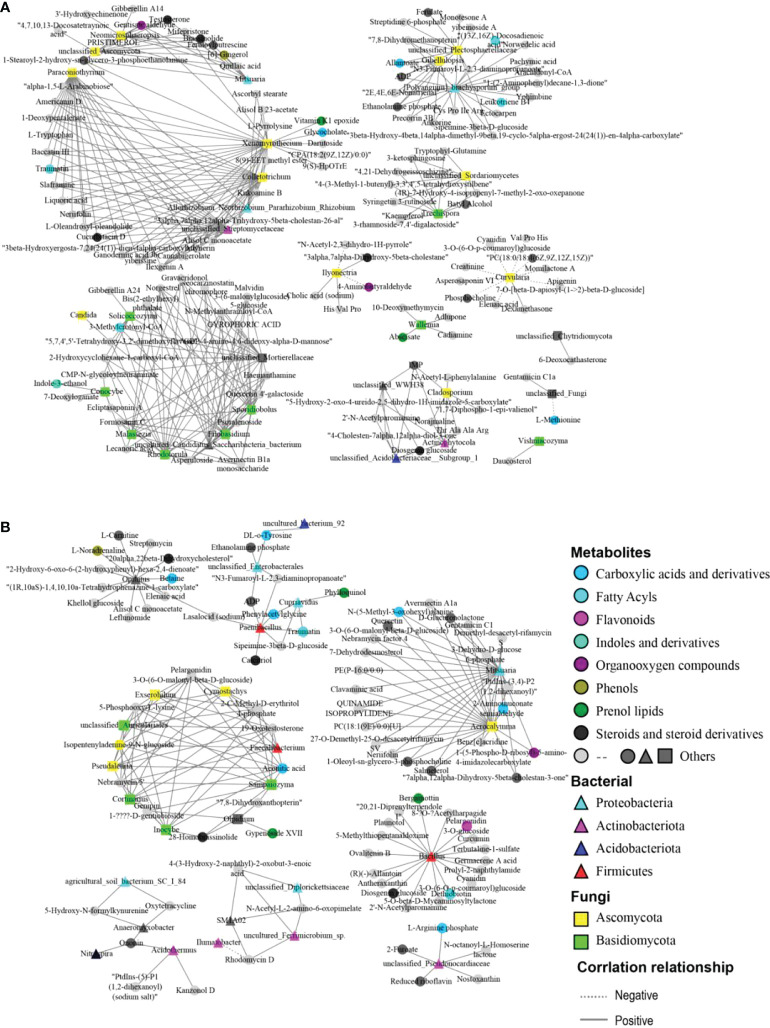
Co-occurrence network analysis among the different microbiota and metabolites from the roots **(A)** and the rhizosphere soils **(B)** of the female and male papaya. The triangles, squares and circles represented bacteria, fungi and metabolites, respectively. Different colors represented the classification of bacteria and fungi at the phylum level and metabolite HMDB classification. The straight line represented a significant positive correlation, while the dotted line represented a negative correlation.

In order to comprehensively explore the possible relationships between differential microorganisms and differential metabolites, co-occurrence network analysis was performed based on Pearson correlation (P< 0.05). The analysis results demonstrated that most differential metabolites were positively correlated with the differential microbes in abundance (**Figure 6**). In the roots of papaya, adynerin was positively correlated with *Paraconiothyrium*, *Colletotrichum*, *Xenomyrothecium*, whereas allantoate exhibited a positive correlation with *Gibellulopsis*, while daucosterol showed a positive relationship with *Vishniacozyma*. Furthermore, norgestrel tended to have a positive association with *Malassezia*, *Rhodotorula*, *Sporidiobolus* and *Filobasidium*. Both gibberellin A14 and gibberellin A24 had a positive correlation with *Neomicrosphaeropsis* and *Solicoccozyma*, respectively. It was also observed that indole-3-ethanol was positively associated with *Conocybe*, while brassinolide and testosterone had a significant positive correlation with *Neomicrosphaeropsis* and *Mitsuaria*.

In the rhizosphere soils, the network revealing the association between metabolites and microorganisms was distinct compared with the roots of the papaya. For instance, 28-Homobrassinolide had a positive correlation with *Olpidium*, *Sampaiozyma* and *Inocybe*, whereas 19-Oxotestosterone revealed a positive association with *Faecalibacterium*, *Cymostachys*, *Exserohilum*, *Cortinarius*, *Pseudaleuria*, and *Sampaiozyma*, while D-Glucuronolactone was positively correlated with *Mitsuaria*, *Acrocalymma*. We also noticed that aconitic acid exhibited apositive relationship with *Faecalibacterium*, *Exserohilum*, while ADP and betaine were significantly positively correlated with *Paenibacillus* and *Opitutus* in abundance, respectively.

## Discussion

From an evolutionary perspective, hermaphroditic plants have both male and female reproductive organs which results in reduction of genetic variation during the largely self-pollination. However, dioecious plants have high genetic variation because their genetic materials of sexual reproduction were from two or more different individuals. They have the advantage of maintaining genetic diversity of species, which is beneficial to their adaption to different environments by natural selection or artificial selection during domestication of crops (Barrett and Hough, 2013; Lei et al., 2017). Although sexual dimorphism in morphology (Decraene and Smets, 1999; Barrett and Hough, 2013), physiology (Laporte and Delph, 1996), timing of development (Gouker et al., 2021) and resistance to stress (Melnikova et al., 2017; Tonnabel et al., 2017; Liu et al., 2021b) has been overwhelmingly reported, the relationship between metabolic profiles and microbial communities in roots and rhizosphere soils of dioecious plants has rarely been investigated. In the present study, we found that sex-based differences exhibited in metabolites and microbiota in dioecious papaya.

For the metabolic composition, the variations in root rhizosphere soils of the males and females were mainly influenced by niches, and the sexual dimorphism exhibited in the metabolic profiles of male and female papaya. Studies have revealed that sex hormones, including testosterone, progesterone, and the other estrogens were associated with sexual differentiation and reproduction (Bouman et al., 2005; Burger, 2006; Rastrelli et al., 2018). Here, differential metabolites in the roots of papaya and some sex hormones were also detected. For example, male hormone such as testosterone was found significantly up-regulated in the male papaya while the female hormone norgestrel showed a significant up-regulation in the female papaya. These results suggested that sex hormones in papaya may also affect sexual differentiation in a dose-dependent manner.

Besides the sex hormones, the phytohormones such as brassinolide, 28-homobrassinolide, jasmonic acid (JA) and GAs were also identified in the roots and rhizosphere soils. Brassinosteroids (BRs) are a group of steroidal plant hormones that interact with auxin and GAs to regulate many developmental processes including stem, petioles and hypocotyls elongation (Yin et al., 2002; Kim et al., 2009). Hence, males seemed to require a singnificant amount of BRs to promote their strong root system, which resulted in the accumulation of brassinolide and 28-homobrassinolide in the roots and rhizosphere soils. In addition, BRs and JAs were reported to mediate the balance between plant growth and defense responses (Liao et al., 2020). The relatively higher abundance of brassinolide, 28-homobrassinolide and JA in both the roots and rhizosphere soils of the male papaya suggested male papaya had higher resistance to stresses.

Plants have mutualistic relationships with their inhabiting microbiome which is referred to as the host’s second or extended genome (Beckers et al., 2017). They interact and evolve with each other over time for nutrient acquisition, productivity and resistance (Hacquard et al., 2015; Beckers et al., 2017; Xiong et al., 2021). Additionally, the male and female papaya have evolved to have preference for some microbes. For instance, male papaya roots or rhizosphere soils enriched some nitrogen-fixing bacteria such as *Allorhizobium-Neorhizobium-Pararhizobium-Rhizobium*, *Brevundimonas* and *Microvirga* (Mousavi et al., 2014; Radl et al., 2014; Naqqash et al., 2020; Pang et al., 2021; Pang et al., 2022b). The enriched testosterone identified in the roots and rhizosphere soils of the male papaya has also been reported to promote nitrogen retention in human e (Bhasin et al., 2003). These results are consistent with the demands of high nitrogen nutrients for numerous pollens in some species (Lei et al., 2017; Tonnabel et al., 2017; Liu et al., 2021b). The roots or rhizoshphere soil of female papaya were enriched with Candidatus, Solibacter and Tumebacillus that utilize organic carbon sources (Baek et al., 2011; Challacombe et al., 2011; Naqqash et al., 2020), which provided evidence that females consume more carbon nutrient for their reproduction leading to fruit production, a major sink organ (Harris and Pannell, 2008; Lei et al., 2017).

Our finding also revealed that the abundance of some fungi genera exhibited distinct distribution patterns in both the male and female papaya roots. For example, *Candida*, *Solicoccozyma* and *Rhodotorula* which play important role in the degradation of a variety of organic materials (Shailubhai et al., 1985; Luo et al., 2021) showed higher abundance in the female papaya roots, suggesting that they could collaborate with endophytic bacteria to provide carbon nutrient for female papaya reproduction. However, in the roots of male papaya, some fungal species such as *Gibellulopsis* (Giraldo and Crous, 2019 *)*, *Curvularia* (Cui et al., 2020), *Colletotrichum* (Gautam, 2014) and *Vishniacozyma* (Pem et al., 2020) which have been described as plant pathogens were enriched in male papaya. It has also been reported that some phytopathogens in the environment are of endophyte origins and that these innocuous fungal endophytes are beneficial to their host plants against the biotic and abiotic stresses through producing secondary metabolites, which is tigger by a long process of co-evolution (Tan and Zou, 2001). Most differential metabolites demonstrated a positive correlation with the differential microbes, suggesting that these differential metabolites detected in the papaya roots had a positive effect on the density and structure of the microbes in roots and rhizosphere soils.

## Data availability statement

The original contributions presented in the study are publicly available. This data can be found here: NCBI, PRJNA857495.

## Author contributions

RM, YZ and ZP designed the research. YZ and ZP performed the experiment and data analysis, and draft the manuscript. RM, ZY and NF supervised the experiment, revised and improved the manuscript. ZP and HJ collected plant materials and extracted DNA. All authors contributed to the article and approved the submitted version.

## Funding

This research was funded by National Science Foundation (NSF) Plant Genome Research Program Award DBI-1546890 to RM.

## Acknowledgments

We thank BMKCloud for providing a platform for data analysis.

## Conflict of interest

The authors declare that the research was conducted in the absence of any commercial or financial relationships that could be construed as a potential conflict of interest.

## Publisher’s note

All claims expressed in this article are solely those of the authors and do not necessarily represent those of their affiliated organizations, or those of the publisher, the editors and the reviewers. Any product that may be evaluated in this article, or claim that may be made by its manufacturer, is not guaranteed or endorsed by the publisher.
