# Supplementary material for: Sex-based metabolic and microbiota differences in roots and rhizosphere soils of dioecious papaya (Carica papaya L.)
Source: Front Plant Sci. 2022 Oct 13;13:991114. doi: 10.3389/fpls.2022.991114 (PMC9612958; doi:10.3389/fpls.2022.991114)
Supplement: Supplementary file 1 [file DataSheet_1.pdf]

Table S1. Comparison of relative abundance of metabolites in each group.

Metabolites	FR	FRh	MR	MRh
Glycerophospholipids	25.3 a	14.8 a	20.3 a	15.7 a
Steroids and steroid derivatives	**17 a**	**20.5 a**	7.4 b	7.5 b
Carboxylic acids and derivatives	9.6 ab	10.4 b	14.1 ab	**15.3 a**
Fatty Acyls	9.1 ab	9.9 b	12.5 a	11.6 ab
Prenol lipids	7.6 a	9 a	9.7 a	9.9 a
Organonitrogen compounds	6.9 a	6.3 a	9.5 a	11.6 a
Macrolides and analogues	4.6 a	5.8 a	8.4 a	10.3 a
Benzene and substituted derivatives	2.6 a	2.8 a	3 a	3.4 a
Cinnamic acids and derivatives	1.1 b	1.6 b	**3.1 a**	**2.9 a**
Depsides and depsidones	0.9 ab	0.5 b	3.5 a	**3.6 a**
Organooxygen compounds	**2.3 a**	**3 a**	0.8 b	0.9 b
Flavonoids	**3.5 a**	**2.5 b**	0 c	0 c
Imidazopyrimidines	1.1 a	1.3 a	2 a	2.3 a
Glycerolipids	0.9 a	1.6 a	1 a	0.6 a
Phenols	0.8 a	0.9 a	1.1 a	0.8 a
Purine nucleosides	0.5 a	0.8 a	0.6 a	0.6 a
Sphingolipids	0.5 a	0.5 a	0.6 a	0.6 a
Coumarins and derivatives	0.5 ab	**1.1 a**	0 b	0 b
Indoles and derivatives	**0.7 a**	**0.7 a**	0 b	0 b
Others	4.4 a	5.8 a	2.4 b	2.3 b

Table S2A. Differential analysis of metabolites in the roots between sexes.

otu_id	logCPM	logFC	PValue	Mean A	Mean B	level	
**neg_6866**	0.00	-10.90	0.00	398.20	0.00	Depleted	(4R)-7-Hydroxy-4-isopropenyl-7-methyl-2-oxo-oxepanone
**pos_1284**	0.03	-1.16	0.00	2374.50	960.83	Depleted	His Val Pro
**pos_7840**	0.00	-6.01	0.00	125.03	1.92	Depleted	Gravacridonol
**pos_7427**	0.00	-1.02	0.00	196.49	87.90	Depleted	Squalene
**pos_40**	0.02	-0.90	0.00	1056.01	516.32	Depleted	Ivacaftor
**pos_9501**	0.02	-1.11	0.00	1194.18	499.45	Depleted	Gibberellin A24
**pos_7946**	0.00	-6.07	0.00	182.66	2.18	Depleted	4-(3-Methyl-1-butenyl)-3,3',4',5-tetrahydroxystilbene
**pos_7505**	0.01	-0.65	0.00	704.04	412.10	Depleted	Hexadecanoic acid
**pos_6131**	0.00	-0.86	0.00	138.90	68.69	Depleted	Erythromycin B
**pos_6544**	0.01	-0.56	0.00	309.09	191.06	Depleted	5,6-DHET
**pos_7225**	0.01	-0.39	0.00	399.54	278.07	Depleted	(9Z)-Hexadecenoic acid
**pos_3565**	0.02	-0.71	0.00	1437.93	804.60	Depleted	Val Pro His
**pos_3510**	0.02	-0.68	0.00	1281.75	727.97	Depleted	Gibberellin A36
**pos_5505**	0.01	-1.44	0.00	383.25	127.03	Depleted	Dihydrourocanate
**pos_6342**	0.01	-0.42	0.00	491.90	333.75	Depleted	8,9-DHET
**pos_5513**	0.01	-1.80	0.00	407.40	104.78	Depleted	Aurantinidin
**pos_5524**	0.02	-2.12	0.00	1241.59	254.43	Depleted	5,7,4',5'-Tetrahydroxy-3,2'-dimethoxyflavone
**pos_6115**	0.01	-0.74	0.00	664.48	364.43	Depleted	(-)-Corey Lactone Diol
**pos_5930**	0.01	-0.70	0.00	567.18	315.05	Depleted	Ecliptasaponin A
**neg_4692**	0.00	-2.39	0.00	313.18	54.44	Depleted	5-Amino-6-(5'-phosphoribosylamino)uracil

**pos_7065**	0.01	-0.58	0.00	522.91	318.89	Depleted	Benzyl cinnamate
**pos_3070**	0.05	-0.69	0.00	3210.90	1809.80	Depleted	2-Oleoyl-1-palmitoyl-sn-glycero-3-phosphocholine
**pos_7022**	0.29	-0.26	0.00	16126.90	12313.68	Depleted	Propyl 2,4-decadienoate
**neg_7000**	0.00	-1.25	0.00	254.40	97.03	Depleted	Asperosaponin VI
**neg_6266**	0.00	-1.87	0.00	79.33	19.24	Depleted	Deacetylvindoline
**neg_4504**	0.00	-4.29	0.00	269.70	13.15	Depleted	3-Isopropenylpimelyl-CoA
**neg_2708**	0.00	-1.92	0.00	315.26	72.76	Depleted	2-epi-5-epi-Valiolone 7-phosphate
**neg_6963**	0.01	-1.78	0.00	559.41	150.60	Depleted	Macrocin
**neg_6415**	0.00	-4.08	0.00	245.10	12.37	Depleted	Haemanthamine
**pos_6773**	0.00	-0.55	0.00	150.86	94.49	Depleted	(1E,4S,5E,7R)-Germacra-1(10),5-dien-11-ol
**pos_9386**	0.11	-0.60	0.00	6939.55	4153.46	Depleted	Phosphocholine
**neg_4188**	0.03	-3.32	0.00	2318.58	211.29	Depleted	GDP-4-amino-4,6-dideoxy-alpha-D-mannose
**pos_8072**	0.00	-3.54	0.00	37.94	3.16	Depleted	6-Deoxocathasterone
**pos_7561**	0.01	-0.40	0.00	775.47	538.90	Depleted	Vindoline
**pos_8566**	0.02	-0.27	0.00	1260.15	948.98	Depleted	Sepiapterin
**pos_1844**	3.86	-1.21	0.00	276936.44	108126.79	Depleted	PC(18:0/18:4(6Z,9Z,12Z,15Z))
**pos_6294**	0.00	-0.98	0.00	53.71	25.26	Depleted	Solavetivol
**pos_4224**	1.10	-0.26	0.00	62120.62	47378.97	Depleted	2-Chloro-L-phenylalanine
**pos_7250**	0.00	-2.51	0.00	133.76	20.69	Depleted	Chenodeoxycholate
**neg_7153**	0.00	-2.60	0.00	416.67	62.20	Depleted	Hydrogenobyrinate a,c diamide
**pos_2402**	0.01	-0.37	0.00	393.72	277.65	Depleted	Octadecanoic acid
**neg_6954**	0.00	-1.61	0.00	342.35	102.77	Depleted	Cholic acid (sodium)

**pos_43**	0.01	-0.90	0.00	358.82	176.12	Depleted	Momilactone A
**pos_7832**	0.00	-0.48	0.00	101.68	66.97	Depleted	Benzofuran
**neg_4521**	0.03	-1.30	0.01	2539.28	929.27	Depleted	Polygalaxanthone III
**neg_6833**	0.00	-1.33	0.01	159.78	57.10	Depleted	7-Deoxyloganate
**pos_6817**	0.16	-0.26	0.01	8973.36	6862.18	Depleted	(±)7-epi Jasmonic Acid
**pos_7866**	0.15	-0.28	0.01	8747.69	6602.56	Depleted	dodecanamide
**neg_6214**	0.00	-4.62	0.01	365.20	13.13	Depleted	N-Methylserotonin
**pos_8573**	0.00	-0.68	0.01	192.54	109.95	Depleted	5-Methylthio-D-ribose
**pos_3832**	0.01	-0.37	0.01	385.51	271.91	Depleted	Isoeugenol
**neg_3603**	0.01	-0.63	0.01	496.16	291.41	Depleted	Cyanidin 3-O-(6-O-p-coumaroyl)glucoside
**pos_5427**	0.01	-0.88	0.01	346.07	171.61	Depleted	5-Hydroxymethyldeoxycytidylate
**pos_6613**	0.00	-1.77	0.01	136.95	35.70	Depleted	3-ketosphingosine
**pos_6842**	0.01	-0.36	0.01	507.79	360.46	Depleted	Norecasantalol
**pos_6452**	0.00	-3.84	0.01	177.18	11.63	Depleted	Norgestrel
**pos_8801**	0.01	-0.52	0.01	373.73	237.10	Depleted	5-Aminopentanamide
**neg_1827**	0.02	-3.02	0.01	2010.83	227.12	Depleted	Tryptophyl-Glutamine
**neg_1766**	0.15	-1.77	0.01	11549.19	2967.23	Depleted	Loganic Acid
**pos_9632**	1.37	-0.48	0.01	82811.75	53827.35	Depleted	Zeaxanthin diglucoside
**pos_4548**	0.00	-0.84	0.01	272.31	141.27	Depleted	(±)-2-(3,4-Dihydroxyphenyl)-1,3-benzodioxole-5-carboxaldehyde
**pos_8558**	0.02	-0.27	0.01	1111.02	842.03	Depleted	DIBOA
**pos_7789**	0.00	-4.11	0.01	26.55	1.26	Depleted	Batyl Alcohol
**pos_6349**	0.00	-0.54	0.01	163.22	101.89	Depleted	Indole-3-ethanol

**neg_3307**	0.02	-1.59	0.01	1519.46	438.51	Depleted	GYROPHORIC ACID
**neg_5967**	0.01	-0.79	0.01	738.52	392.39	Depleted	(S)-Coclaurine
**pos_5297**	0.00	-1.87	0.01	151.82	37.42	Depleted	(2S)-5,7,3',4'-Tetrahydroxyflavanone 7-glucuronide
**pos_1640**	0.01	-1.76	0.01	783.33	203.75	Depleted	Engeletin
**pos_8074**	0.02	-0.36	0.01	1005.68	711.03	Depleted	5-Amino-5-(4-hydroxybenzyl)-6-(D-ribitylimino)-5,6-dihydrouracil
**pos_7634**	0.08	-0.26	0.01	4539.74	3463.48	Depleted	Ethyl salicylate
**pos_7278**	0.00	-0.32	0.01	191.26	139.92	Depleted	(5S,6S)-6-Amino-5-hydroxycyclohexa-1,3-diene-1-carboxylate
**pos_3077**	0.04	-0.24	0.01	2133.83	1652.90	Depleted	2'-O-Methyluridine
**pos_3091**	0.03	-0.18	0.01	1543.55	1242.69	Depleted	Alanopine
**neg_6212**	0.00	-0.63	0.01	198.32	117.94	Depleted	N-Methyltryptamine
**pos_2122**	0.01	-0.25	0.01	411.42	314.40	Depleted	Diphenylamine
**pos_6798**	0.00	-0.50	0.02	77.14	49.92	Depleted	L-Methionine
**neg_4327**	0.01	-0.85	0.02	987.14	501.96	Depleted	Apigenin 7-O-[beta-D-apiosyl-(1->2)-beta-D-glucoside]
**pos_5558**	0.15	-0.72	0.02	9589.33	5278.26	Depleted	Phenylacetylglutamine
**pos_7848**	0.02	-0.21	0.02	1265.96	996.50	Depleted	Alanyl-Isoleucine
**pos_7834**	0.02	-0.20	0.02	1232.32	980.38	Depleted	p-Coumaric acid
**pos_4534**	0.04	-0.70	0.02	2371.61	1352.36	Depleted	Sulfamerazine
**pos_3036**	0.02	-0.27	0.02	922.28	699.37	Depleted	Safranal
**pos_6930**	0.04	-0.39	0.02	2446.71	1706.07	Depleted	Creatinine
**pos_2563**	0.05	-0.82	0.02	3123.90	1591.77	Depleted	Bis(2-ethylhexyl) phthalate
**pos_6339**	0.04	-0.21	0.02	2388.54	1881.42	Depleted	Nortropine
**pos_7835**	0.02	-0.21	0.02	1150.20	908.29	Depleted	Precocene II

**pos_6441**	0.00	-0.71	0.02	43.86	24.63	Depleted	beta-PC-M6
**neg_4419**	0.02	-4.04	0.02	1761.32	102.23	Depleted	N-Methylanthraniloyl-CoA
**pos_7471**	0.00	-0.70	0.02	59.06	33.26	Depleted	(R)-(-)-Mellein
**pos_6622**	0.00	-3.87	0.02	223.13	13.50	Depleted	Quercetin 4'-galactoside
**pos_4923**	0.00	-0.67	0.02	197.60	114.36	Depleted	Reduced coenzyme F420
**pos_4098**	1.69	-0.23	0.02	94470.68	73735.09	Depleted	Indoxyl
**pos_6515**	0.01	-1.08	0.02	604.77	257.27	Depleted	Narcotine hemiacetal
**pos_7053**	0.01	-0.26	0.02	323.59	246.75	Depleted	(S)-Limonene
**neg_3231**	0.01	-0.65	0.02	601.87	349.45	Depleted	GDP-valienol
**neg_2635**	0.02	-4.86	0.02	1526.88	44.56	Depleted	2-Hydroxycyclohexane-1-carboxyl-CoA
**neg_1736**	0.01	-0.99	0.02	558.92	250.24	Depleted	Lecanoric acid
**pos_7601**	0.01	-0.23	0.02	515.69	402.10	Depleted	3-[4-Hydroxy-3-(3-methyl-2-butenyl)phenyl]-2-propenal
**pos_2350**	0.04	-0.27	0.02	2022.12	1531.50	Depleted	1-Stearoyl-rac-glycerol
**pos_2405**	0.08	-0.29	0.02	4640.11	3479.23	Depleted	Hexadecyl Acetyl Glycerol
**neg_2920**	0.00	-2.67	0.02	396.42	54.84	Depleted	Neocarzinostatin chromophore
**pos_5641**	0.02	-1.21	0.02	1466.62	576.09	Depleted	Syringetin 3-rutinoside
**pos_5873**	0.00	-0.87	0.02	67.33	32.68	Depleted	Histidinyl-Valine
**pos_3776**	0.00	-0.53	0.02	144.05	90.97	Depleted	1-Hydroxyphenazine
**pos_6623**	0.00	-4.46	0.02	157.94	6.38	Depleted	Asperuloside
**pos_7294**	0.00	-0.42	0.02	169.34	115.48	Depleted	1D-1-Guanidino-3-amino-1,3-dideoxy-scyllo-inositol
**neg_4499**	0.02	-0.74	0.02	1369.68	753.85	Depleted	8-Demethyl-8-(2-O-methyl-alpha-L-rhamnosyl)tetracenomycin C
**pos_2762**	0.09	-0.24	0.02	5320.18	4116.14	Depleted	Sphingosyl-phosphocholine

**pos_6772**	0.03	-0.23	0.02	1612.37	1261.24	Depleted	Vulgarone A
**pos_8556**	0.03	-0.20	0.02	1536.24	1216.39	Depleted	Salicylic acid
**pos_4949**	0.01	-0.55	0.02	464.74	292.87	Depleted	Leucodelphinidin
**pos_6512**	0.00	-4.65	0.02	296.63	11.20	Depleted	Avermectin B1a monosaccharide
**pos_6951**	0.01	-0.27	0.02	589.17	446.38	Depleted	Pseudoionone
**pos_3482**	0.00	-0.29	0.02	249.70	185.66	Depleted	Thiourocanic acid
**pos_2949**	0.01	-2.51	0.02	787.02	123.64	Depleted	DG(16:0/17:2(9Z,12Z)/0:0)[iso2]
**pos_7036**	0.01	-0.26	0.02	362.38	275.70	Depleted	7-Deoxyloganetin
**neg_5461**	0.00	-1.43	0.02	315.87	106.80	Depleted	Novobiocin
**pos_4073**	0.02	-0.27	0.02	1306.39	986.14	Depleted	Benzaldehyde
**pos_3105**	0.01	-0.28	0.02	570.95	430.01	Depleted	Pentoxifylline
**pos_8192**	0.01	-0.88	0.02	732.27	370.02	Depleted	22-Hydroxydocosanoate
**pos_7812**	0.00	-0.33	0.02	186.66	135.50	Depleted	(E)-4-stilbenol
**pos_6653**	0.00	-3.08	0.03	30.59	3.52	Depleted	Psoralenoside
**pos_8478**	0.02	-0.26	0.03	1364.32	1047.94	Depleted	19-Hydroxytestosterone
**neg_5721**	0.00	-1.84	0.03	90.68	24.40	Depleted	Prostaglandin G2
**pos_4966**	0.00	-1.03	0.03	160.89	71.49	Depleted	dTDP-3-amino-2,3,6-trideoxy-4-keto-D-glucose
**pos_2661**	0.00	-0.35	0.03	199.99	142.49	Depleted	Tropine
**neg_3589**	0.00	-1.97	0.03	227.00	51.28	Depleted	3-Oxohexanoyl-CoA
**pos_5080**	0.00	-1.41	0.03	331.36	116.86	Depleted	(3S)-Citramalyl-CoA
**neg_4648**	0.01	-0.92	0.03	390.13	190.16	Depleted	Deacetylasperulosidic Acid
**neg_7189**	0.03	-0.90	0.03	1730.90	829.71	Depleted	Hydroxychlorobactene glucoside

**pos_2658**	0.00	-0.27	0.03	213.10	160.82	Depleted	Pseudopelletierine
**pos_5300**	0.01	-1.25	0.03	366.08	144.53	Depleted	Itaconyl-CoA
**pos_6984**	0.01	-0.38	0.03	406.46	285.08	Depleted	Palmitoleamide
**pos_6996**	0.00	-0.55	0.03	168.41	106.07	Depleted	Stizolobate
**pos_8025**	0.01	-0.48	0.03	385.96	255.37	Depleted	(6Z,9Z,12Z)-Octadecatrienoic acid
**neg_1767**	0.01	-1.88	0.03	633.00	150.65	Depleted	Deoxynogalonate
**neg_3647**	0.02	-1.08	0.03	1158.23	494.49	Depleted	Taxifolin
**neg_6680**	0.01	-1.18	0.03	410.51	163.45	Depleted	3,4-Dihydroxy-9,10-secoandrosta-1,3,5(10)-triene-9,17-dione
**pos_8210**	0.00	-0.98	0.03	44.92	20.20	Depleted	3alpha,7alpha-Dihydroxy-5beta-cholestane
**pos_7428**	0.00	-0.58	0.03	253.15	154.84	Depleted	Pro Lys Arg His
**neg_4049**	0.00	-2.99	0.03	270.84	31.56	Depleted	S-Glutathionyl-L-cysteine
**pos_7476**	0.02	-0.34	0.03	1184.63	859.38	Depleted	Dehydrooreadone
**pos_8028**	0.03	-0.21	0.03	1770.79	1401.24	Depleted	Cycloionone
**pos_2511**	0.00	-0.37	0.03	250.95	175.72	Depleted	D-Glucosamine 6-phosphate
**pos_5408**	0.00	-1.29	0.03	144.52	53.99	Depleted	Cyanidin 3-O-beta-D-sambubioside
**pos_8591**	0.01	-0.25	0.03	342.38	264.02	Depleted	6-Hydroxydopamine hydrochloride
**neg_7503**	0.17	-1.05	0.03	11706.94	5229.39	Depleted	Solanine
**pos_8557**	0.08	-0.21	0.03	4494.75	3548.27	Depleted	4-Hydroxyphenylglyoxylate
**neg_4533**	0.00	-3.48	0.03	300.41	25.18	Depleted	3-Methylcrotonyl-CoA
**neg_3548**	0.01	-1.51	0.03	616.53	191.12	Depleted	CMP-N-glycoloylneuraminate
**neg_3621**	0.01	-1.02	0.03	620.77	283.29	Depleted	UDP-N-acetyl-3-(1-carboxyvinyl)-D-glucosamine
**pos_3497**	0.10	-0.21	0.03	5579.23	4416.69	Depleted	(S)-5-Oxo-2,5-dihydrofuran-2-acetate

**neg_6235**	0.02	-4.20	0.03	2228.89	115.57	Depleted	Formosanin C
**pos_5299**	0.03	-2.72	0.03	2244.74	302.26	Depleted	Malvidin 3-(6-malonylglucoside) 5-glucoside
**pos_5526**	0.00	-4.82	0.03	20.03	0.62	Depleted	S-Methyl-5-thio-D-ribose 1-phosphate
**neg_5870**	0.00	-0.78	0.03	114.61	61.00	Depleted	D-Glucosamine
**neg_7427**	0.02	-1.39	0.03	1193.31	433.54	Depleted	Mycobactin S
**pos_4959**	0.02	-0.47	0.04	995.07	655.57	Depleted	Torvanol A
**pos_2914**	0.02	-0.16	0.04	1175.80	961.83	Depleted	Methionyl-Glutamate
**pos_7182**	0.00	-0.47	0.04	78.03	51.24	Depleted	5-Oxoproline
**pos_7638**	0.01	-0.25	0.04	480.74	367.91	Depleted	Phenol
**pos_7070**	0.27	-0.26	0.04	15253.01	11626.30	Depleted	Volicitin
**pos_5129**	0.02	-1.46	0.04	1522.76	505.65	Depleted	Kaempferol 3-rhamnoside-7,4'-digalactoside
**pos_5153**	0.00	-1.45	0.04	72.58	24.70	Depleted	L-Glutamyl 5-phosphate
**neg_6625**	0.01	-1.88	0.04	404.90	99.95	Depleted	Phosphatidylethanolamine 16_1-18_1
**neg_7492**	0.29	-0.78	0.04	18717.65	9708.24	Depleted	10beta,14beta-Dihydroxytaxa-4(20),11-dien-5alpha-yl acetate
**pos_4930**	0.01	-1.63	0.04	706.07	216.44	Depleted	(S)-3-Hydroxyoctanoyl-CoA
**pos_7320**	0.03	-0.21	0.04	1583.83	1254.79	Depleted	Ethyl 3-phenylpropanoate
**pos_8560**	0.07	-0.23	0.04	3756.55	2933.93	Depleted	Anofinic acid
**pos_6814**	0.01	-0.23	0.04	304.58	237.07	Depleted	2-Isopropyl-1,4-benzenediol
**pos_5017**	0.01	-0.43	0.04	547.19	368.88	Depleted	Elenaic acid
**neg_4614**	0.20	-0.87	0.04	13634.36	6748.74	Depleted	Theaflavin
**neg_4438**	0.00	-1.76	0.04	336.95	90.55	Depleted	Sulfazecin
**pos_2788**	0.01	-0.24	0.04	306.72	235.28	Depleted	5,2'-O-dimethyluridine

**pos_6703**	0.00	-0.85	0.04	175.57	89.37	Depleted	Stachyose
**pos_8361**	0.10	-0.18	0.04	5573.25	4493.05	Depleted	L-Palmitoylcarnitine
**pos_6976**	0.01	-0.31	0.04	853.07	630.00	Depleted	Linoleoyl ethanolamide
**pos_5328**	0.00	-0.28	0.04	261.25	196.72	Depleted	5-(3'-Carboxy-3'-oxopropenyl)-4,6-dihydroxypicolinate
**pos_6293**	0.00	-0.68	0.04	38.74	21.99	Depleted	Phthalate 3,4-cis-dihydrodiol
**neg_3505**	0.00	-1.90	0.04	81.36	19.98	Depleted	5'-Phosphoribosylglycinamide
**pos_3687**	0.00	-1.46	0.04	87.42	27.73	Depleted	Cytidine 5'-monophosphate (CMP)
**pos_6556**	0.00	-0.34	0.05	127.73	91.43	Depleted	1-Deoxy-11beta-hydroxypentalenate
**neg_1988**	0.00	-0.67	0.05	270.85	153.67	Depleted	Ajmaline
**pos_8180**	0.10	-0.14	0.05	5543.49	4587.06	Depleted	5-Aminopentanoic acid
**pos_8073**	0.00	-0.43	0.05	142.09	95.58	Depleted	4-Methoxy-2,2'-bipyrrole-5-carbaldehyde
**pos_7620**	0.01	-0.21	0.05	499.07	393.77	Depleted	Histidinyl-Isoleucine
**pos_5506**	0.00	-2.68	0.05	153.28	21.84	Depleted	4,21-Dehydrogeissoschizine
**neg_4762**	0.02	-0.55	0.05	1036.61	641.14	Depleted	Ellagic acid
**pos_7550**	0.00	-0.28	0.05	237.13	177.71	Depleted	4-(4-Methyl-3-pentenyl)-3-cyclohexene-1-carboxaldehyde
**pos_2281**	0.00	-0.26	0.05	155.62	118.17	Depleted	5-Ethyl-2-methylpyridine
**neg_7347**	0.00	18.91	0.00	0.00	27.29	Enriched	[6]-Gingerol
**pos_6545**	0.00	7.98	0.00	1.67	297.72	Enriched	Cadiamine
**pos_8750**	0.01	3.74	0.00	51.62	599.99	Enriched	yibeinoside A
**pos_8815**	0.08	2.62	0.00	1225.99	7011.67	Enriched	Glyuranolide
**neg_7246**	0.03	3.11	0.00	327.68	2620.21	Enriched	Calcitetrol
**neg_5769**	0.09	2.78	0.00	1309.10	8027.41	Enriched	Streptidine 6-phosphate

**pos_8814**	0.15	2.09	0.00	2918.51	11658.04	Enriched	PRISTIMEROL
**neg_7297**	0.00	7.88	0.00	1.05	206.39	Enriched	Taurocholate
**pos_7421**	0.00	4.45	0.00	9.21	197.11	Enriched	Alisol B 23-acetate
**pos_9581**	0.03	2.12	0.00	626.24	2425.50	Enriched	Gentamicin C2
**pos_6590**	0.00	1.95	0.00	37.85	132.46	Enriched	2-Hexaprenyl-6-methoxy-1,4-benzoquinone
**pos_9579**	0.02	0.67	0.00	908.90	1307.01	Enriched	Gentamicin C1a
**pos_8934**	0.12	1.86	0.00	2795.75	9199.15	Enriched	Cholic Acid Methyl Ester
**pos_9583**	0.01	1.75	0.00	306.38	931.62	Enriched	Avermectin A1a aglycone
**pos_6860**	0.03	2.53	0.00	557.31	2889.18	Enriched	Ferulate
**pos_5066**	0.03	1.27	0.00	1075.54	2330.72	Enriched	Thr Ile Ser Val
**pos_9076**	0.62	0.76	0.00	24191.80	37593.04	Enriched	Spheroidenone
**neg_7293**	0.00	3.99	0.00	11.88	169.04	Enriched	Sapindoside A
**neg_2266**	0.01	7.49	0.00	5.78	857.34	Enriched	Anamorelin
**pos_8906**	0.00	3.01	0.00	7.67	55.43	Enriched	Anatalline
**pos_4373**	0.03	1.82	0.00	618.37	1975.57	Enriched	3-O-Methylisoetharine
**pos_8868**	0.01	1.89	0.00	319.69	1127.54	Enriched	4alpha-Methyl-5alpha-ergosta-8,14,24(28)-trien-3beta-ol
**pos_8927**	0.00	1.15	0.00	35.36	71.26	Enriched	Lysyl-Proline
**pos_7451**	0.00	7.07	0.00	0.00	21.72	Enriched	2'-N-Acetylparomamine
**pos_8807**	0.00	1.64	0.00	30.06	86.74	Enriched	1D-1-Guanidino-1-deoxy-3-dehydro-scyllo-inositol
**neg_7229**	0.46	3.07	0.00	5221.83	40811.14	Enriched	His Thr Lys Lys
**pos_8565**	0.00	10.35	0.00	0.32	461.45	Enriched	alpha-1,5-L-Arabinobiose
**neg_6233**	0.02	1.91	0.00	360.29	1289.58	Enriched	Mifepristone

**pos_2107**	0.00	1.33	0.00	75.58	174.78	Enriched	Paspalicine
**neg_5525**	0.00	7.30	0.00	0.00	25.39	Enriched	5-Hydroxy-2-oxo-4-ureido-2,5-dihydro-1H-imidazole-5-carboxylate
**pos_5684**	0.00	1.58	0.00	55.24	150.70	Enriched	5-Oxopentanoate
**pos_8537**	0.00	10.90	0.00	0.04	348.51	Enriched	Americanin D
**pos_2749**	0.20	1.01	0.00	6917.02	12938.60	Enriched	PG(18_2(9Z,12Z)_18_0)
**pos_9475**	0.03	0.93	0.00	1210.62	2141.80	Enriched	26-Hydroxybrassinolide
**neg_4583**	0.02	1.47	0.00	698.26	1744.36	Enriched	Berberine
**neg_6524**	0.01	2.08	0.00	243.80	942.14	Enriched	Ganoderenic acid A
**pos_5748**	0.00	2.45	0.00	10.42	50.23	Enriched	5'-Benzoylphosphoadenosine
**neg_7092**	0.02	3.95	0.00	101.92	1532.11	Enriched	Kukoamine B
**neg_4557**	0.01	2.22	0.00	213.49	899.97	Enriched	Byakangelicin
**pos_6962**	0.00	1.56	0.00	62.91	171.72	Enriched	(-)-Jasmonic acid
**neg_7311**	0.01	5.22	0.00	36.87	1259.72	Enriched	Diosgenin glucoside
**pos_8928**	0.01	0.63	0.00	354.58	505.76	Enriched	L-Pyrrolysine
**neg_3465**	0.00	5.66	0.00	1.78	71.68	Enriched	1,7-Diphospho-1-epi-valienol
**pos_4716**	0.00	0.91	0.00	71.87	124.49	Enriched	Vanillylamine
**pos_9594**	0.01	0.90	0.00	367.54	615.56	Enriched	Cyclooctatin
**neg_7231**	0.01	2.93	0.00	178.16	1273.34	Enriched	Cucurbitacin B
**pos_2145**	0.04	0.55	0.00	1661.61	2209.41	Enriched	Thiothece-474
**pos_5227**	0.01	1.07	0.00	503.45	955.84	Enriched	Aldosterone
**pos_2039**	0.03	0.89	0.00	1206.27	2030.45	Enriched	Erythromycin D
**pos_8925**	0.00	1.17	0.00	52.46	108.56	Enriched	Acetyltropine

**pos_8373**	0.01	2.13	0.00	184.90	738.67	Enriched	Adlupone
**neg_7497**	0.01	2.91	0.00	84.30	557.59	Enriched	4-Cholesten-7alpha,12alpha-diol-3-one
**pos_7680**	0.00	2.83	0.00	41.75	273.26	Enriched	Testosterone
**pos_9124**	0.01	3.48	0.00	76.15	731.39	Enriched	Sipeimine-3beta-D-glucoside
**pos_8929**	0.02	0.54	0.00	686.70	913.82	Enriched	3β-hydroxy-estra-5,7,9-trien-17-one
**pos_8945**	0.00	0.72	0.00	177.06	268.09	Enriched	4,7,10,13-Docosatetraynoic acid
**neg_7298**	0.00	8.05	0.00	0.00	43.05	Enriched	10-Deoxysarpagine
**neg_6771**	0.02	1.95	0.00	365.42	1369.20	Enriched	Traumatin
**pos_8920**	0.00	0.95	0.00	173.90	305.49	Enriched	Phenylheptatriyne
**neg_6537**	0.02	2.07	0.00	457.94	1843.41	Enriched	Darutoside
**pos_8017**	0.00	3.40	0.00	19.98	188.25	Enriched	Daucosterol
**pos_1329**	0.00	0.97	0.00	38.62	68.89	Enriched	3-Isochromanone
**neg_6931**	0.00	4.72	0.00	10.58	281.01	Enriched	Slaframine
**pos_7738**	0.04	2.17	0.00	692.33	3102.15	Enriched	Liquoric acid
**pos_2031**	0.23	0.88	0.00	8476.43	14723.71	Enriched	1-Deoxypentalenate
**pos_9523**	0.04	1.19	0.00	1298.82	2754.90	Enriched	Quillaic acid
**pos_8808**	0.01	1.14	0.00	167.99	338.44	Enriched	Histidinyl-Lysine
**pos_7671**	0.00	4.17	0.00	7.48	118.71	Enriched	Pachymic acid
**pos_8956**	0.01	0.76	0.00	396.71	625.98	Enriched	Valyl-Lysine
**pos_8388**	0.00	9.26	0.00	0.00	98.26	Enriched	ADP
**pos_6580**	0.01	4.32	0.00	45.47	895.48	Enriched	Neriifolin
**pos_2528**	0.00	2.38	0.00	28.73	136.80	Enriched	CDP-2,3-bis-O-(geranylgeranyl)-sn-glycerol

**neg_4257**	0.01	4.76	0.00	30.93	830.14	Enriched	L-Tryptophan
**pos_5412**	0.00	1.35	0.00	31.52	73.25	Enriched	d-Dethiobiotin
**neg_6343**	0.02	2.71	0.00	223.57	1456.06	Enriched	L-Oleandrosyl-oleandolide
**pos_8016**	0.01	1.96	0.00	300.09	1132.48	Enriched	Ganodermic acid Jb
**neg_1421**	0.01	1.96	0.00	111.93	399.90	Enriched	Violaceinic acid
**neg_3806**	0.00	3.74	0.00	4.87	57.85	Enriched	Cetoniacytone A
**pos_9245**	0.00	7.30	0.01	2.57	335.24	Enriched	IMP
**neg_5666**	0.00	1.28	0.01	111.77	246.50	Enriched	5-Amino-5-deoxy-3-dehydroshikimate
**neg_5659**	0.02	1.90	0.01	479.35	1629.12	Enriched	Picroside III
**pos_9173**	0.00	1.15	0.01	157.79	316.36	Enriched	20,21-Diprenylterpendole C
**pos_5714**	0.15	1.54	0.01	4206.55	11052.09	Enriched	Isorhamnetin-3-O-glucoside
**pos_1222**	0.00	0.72	0.01	87.46	132.13	Enriched	Isoprene
**pos_1365**	0.08	1.10	0.01	2815.69	5475.92	Enriched	Vidarabine
**neg_6392**	0.00	1.99	0.01	68.22	257.92	Enriched	yibeissine
**pos_9141**	0.00	1.75	0.01	76.10	234.47	Enriched	3'-Hydroxyechinenone
**pos_8383**	0.00	8.51	0.01	0.00	58.29	Enriched	Ethanolamine phosphate
**pos_4591**	0.00	1.16	0.01	31.68	63.33	Enriched	D-Lysopine
**pos_9101**	0.00	2.51	0.01	25.00	123.37	Enriched	Avermectin B1a aglycone
**pos_3976**	0.00	1.24	0.01	156.48	333.24	Enriched	5-(N-Methyl-4,5-dihydro-1H-pyrrol-2-yl)pyridin-2-ol
**pos_5704**	0.00	0.96	0.01	166.80	295.94	Enriched	S-Adenosyl-L-methionine
**pos_5275**	0.00	3.73	0.01	16.38	199.78	Enriched	Dihydrozeatin riboside
**neg_6549**	0.00	3.38	0.01	43.30	421.65	Enriched	1-Stearoyl-2-hydroxy-sn-glycero-3-phosphoethanolamine

**pos_6606**	0.04	1.12	0.01	1202.36	2348.69	Enriched	28-Homobrassinolide
**neg_6397**	0.00	1.75	0.01	79.46	254.47	Enriched	Glycocholate
**pos_7757**	0.00	1.38	0.01	25.75	62.44	Enriched	6-Keto-prostaglandin E1
**pos_5468**	0.00	1.02	0.01	18.04	33.31	Enriched	4-Amino-5-aminomethyl-2-methylpyrimidine
**neg_7217**	0.12	0.64	0.01	5016.98	7016.26	Enriched	Ribulose 1,5-diphosphate
**neg_6588**	0.10	2.07	0.01	2059.06	8361.52	Enriched	3alpha,7alpha,12alpha-Trihydroxy-5beta-cholestan-26-al
**neg_6746**	0.10	0.64	0.01	4153.32	5803.01	Enriched	1-Hydroxy-2-methyl-2-butenyl 4-diphosphate
**pos_8003**	0.00	1.08	0.01	86.55	169.98	Enriched	1-(3,4-Dihydroxyphenyl)-5-hydroxy-3-decanone
**pos_8823**	0.00	1.57	0.01	50.03	137.94	Enriched	Prometryn
**pos_6178**	0.00	1.39	0.01	111.65	265.77	Enriched	2-Propenal, 3-(1,3-benzodioxol-5-yl)-
**neg_6578**	0.03	1.68	0.01	732.37	2207.64	Enriched	Brassinolide
**pos_1983**	0.08	0.34	0.01	3763.75	4341.22	Enriched	Vitamin D3 glucosiduronate
**neg_5361**	0.00	5.23	0.01	11.75	407.21	Enriched	Arachidonyl-CoA
**pos_5980**	0.00	0.48	0.01	187.93	237.95	Enriched	L-Cys-Gly
**pos_4243**	0.42	1.06	0.01	14502.45	27061.66	Enriched	Thr Ala Ala Arg
**pos_3851**	0.00	3.93	0.02	2.61	32.18	Enriched	Gibberellin A14
**pos_9194**	0.02	0.40	0.02	1029.02	1247.95	Enriched	Tetrahydrobiopterin
**neg_4745**	0.01	1.02	0.02	284.59	524.95	Enriched	dTDP-4-amino-4,6-dideoxy-D-glucose
**neg_7433**	0.05	1.35	0.02	1493.61	3580.50	Enriched	16-Feruloyloxypalmitate
**pos_1378**	0.00	6.84	0.02	0.00	20.01	Enriched	Gentisate aldehyde
**pos_9079**	0.03	0.67	0.02	1386.68	1996.90	Enriched	3,29-Dibenzoyl Rarounitriol
**pos_8436**	0.01	1.36	0.02	155.74	373.59	Enriched	Ascorbyl stearate

**pos_8719**	0.00	2.77	0.02	6.90	45.32	Enriched	Paromomycin
**neg_5883**	0.00	2.39	0.02	69.84	335.28	Enriched	(-)-Jasmonoyl-L-isoleucine
**pos_4019**	0.00	2.86	0.02	23.73	162.73	Enriched	His Tyr Val Arg
**neg_3627**	0.00	2.11	0.02	71.00	280.25	Enriched	Allantoate
**neg_5237**	0.05	2.17	0.02	951.45	3851.41	Enriched	Malonylapiin
**neg_1463**	0.01	1.49	0.02	172.15	432.97	Enriched	(2R)-2-Hydroxy-2-methylbutanenitrile
**pos_5785**	0.00	2.37	0.02	21.28	99.43	Enriched	2E,4E,6E-Nonatrienal
**pos_6112**	0.02	0.85	0.02	609.39	1016.62	Enriched	2-Methyl-1,3-cyclohexadiene
**pos_3066**	0.01	0.78	0.02	486.58	765.25	Enriched	DG(18:3(9Z,12Z,15Z)/16:0/0:0)
**neg_4308**	0.00	1.85	0.02	92.33	304.86	Enriched	10-Hydroxydihydrosanguinarine
**pos_8372**	0.00	1.64	0.02	101.15	287.03	Enriched	10-Deoxymethymycin
**neg_1709**	0.01	0.80	0.02	496.00	790.12	Enriched	6-O-Methylguanine
**pos_2236**	0.00	0.32	0.02	193.57	220.58	Enriched	4,8,12,16-hexadecatetraenoic acid
**pos_6681**	0.01	0.88	0.02	329.20	540.29	Enriched	Monotesone A
**pos_3952**	0.00	1.60	0.02	44.54	124.14	Enriched	Estradiol-17beta
**pos_5780**	0.01	2.54	0.02	155.02	816.45	Enriched	7,8-Dihydromethanopterin
**pos_1524**	0.01	1.31	0.02	249.52	548.45	Enriched	Linamarin
**pos_9543**	0.00	1.22	0.02	99.84	207.63	Enriched	Kanamycin A
**pos_9306**	0.01	1.35	0.02	163.15	367.60	Enriched	1-(2-Aminophenyl)decane-1,3-dione
**pos_799**	0.04	1.04	0.02	1465.21	2783.50	Enriched	N-Acetyl-2,3-dihydro-1H-pyrrole
**neg_3951**	0.00	2.09	0.02	60.30	230.96	Enriched	8-Amino-8-demethylriboflavin
**neg_7100**	0.02	1.47	0.02	505.30	1355.57	Enriched	Alisol C monoacetate

**pos_2466**	0.08	0.65	0.02	3426.70	4833.33	Enriched	beta-Carotenone
**pos_6770**	0.01	1.44	0.02	287.47	694.39	Enriched	3beta-Hydroxy-4beta,14alpha-dimethyl-9beta,19-cyclo-5alpha-ergost-24(24(1))-en-4alpha-carboxylate
**pos_4394**	0.02	1.87	0.03	401.04	1351.38	Enriched	Ser Ile Val Leu
**pos_9539**	0.00	0.54	0.03	52.91	70.14	Enriched	N-Acetylserotonin
**pos_5693**	0.04	0.84	0.03	1368.83	2229.22	Enriched	Aloesol
**pos_8387**	0.00	4.33	0.03	9.97	168.89	Enriched	N3-Fumaroyl-L-2,3-diaminopropanoate
**pos_2531**	0.00	0.73	0.03	92.74	141.88	Enriched	(±)8(9)-EET methyl ester
**pos_6955**	0.00	1.91	0.03	51.28	176.83	Enriched	Sojagol
**neg_2202**	0.06	0.49	0.03	2642.48	3425.75	Enriched	Taxa-4(20),11(12)-dien-5alpha-acetoxy-10beta-ol
**pos_5751**	0.00	1.19	0.03	55.74	116.19	Enriched	2-(Hydroxymethyl)-4-oxobutanoate
**pos_4004**	0.01	2.89	0.03	73.91	508.26	Enriched	Cys Pro Ile Arg
**pos_2782**	0.05	1.03	0.03	1599.48	3100.00	Enriched	29-Norcycloartane-3,24-dione
**pos_1336**	0.00	1.34	0.03	71.18	166.46	Enriched	O-beta-D-Xylosylzeatin
**pos_7692**	0.00	3.36	0.03	5.16	48.39	Enriched	Cyasterone
**pos_1510**	0.03	0.80	0.03	1082.92	1716.11	Enriched	Dexamethasone
**neg_6665**	0.01	2.72	0.03	202.67	1270.56	Enriched	Feruloylputrescine
**pos_5725**	0.03	1.89	0.03	621.68	2098.33	Enriched	Amarogentin
**pos_4439**	0.00	1.39	0.03	24.93	59.64	Enriched	Formylisoglutamine
**pos_4266**	0.02	1.98	0.03	487.26	1716.28	Enriched	Norajmaline
**neg_6583**	0.00	2.50	0.03	50.35	274.20	Enriched	Ilexgenin A
**pos_857**	0.00	1.22	0.03	30.39	64.44	Enriched	L-Prolinamide
**pos_6713**	0.00	1.25	0.03	34.55	72.78	Enriched	(13Z,16Z)-Docosadienoic acid

**pos_7245**	0.00	3.09	0.03	21.69	164.19	Enriched	Yohimbine
**pos_7750**	0.02	1.03	0.03	535.71	1041.32	Enriched	9(S)-HpOTrE
**pos_3826**	0.00	1.51	0.03	49.81	129.35	Enriched	Prednisolone Acetate
**neg_4258**	0.00	1.36	0.03	47.35	107.49	Enriched	N-Acetyl-L-phenylalanine
**pos_815**	0.01	0.91	0.03	207.98	359.07	Enriched	S-Sulfanylglutathione
**neg_6282**	0.04	2.24	0.03	671.60	2820.45	Enriched	beta-Paxitriol
**pos_5359**	0.00	2.07	0.03	45.41	176.10	Enriched	Gln Ile Ile
**pos_5675**	0.00	0.97	0.03	91.49	163.80	Enriched	Tartronate semialdehyde
**pos_3994**	0.00	1.45	0.03	66.60	168.38	Enriched	gamma-Glutamyltyramine
**neg_6103**	0.00	2.89	0.03	33.53	243.74	Enriched	Vitamin K1 epoxide
**neg_5381**	0.01	1.04	0.03	222.92	423.69	Enriched	Guanidinoproclavaminic acid
**pos_5678**	0.01	1.74	0.04	215.84	655.91	Enriched	UDP-N-acetylmuramoyl-L-alanyl-gamma-D-glutamyl-meso-2,6-diaminopimelate
**pos_5793**	0.01	4.18	0.04	39.21	631.42	Enriched	Precorrin 3B
**pos_2232**	0.00	0.75	0.04	28.02	43.06	Enriched	4-Amino-5-hydroxymethyl-2-methylpyrimidine
**pos_3825**	0.03	0.84	0.04	1079.68	1756.04	Enriched	Glutathionylspermidine
**neg_4403**	0.24	1.13	0.04	7930.98	15584.12	Enriched	Norfloxacin
**pos_8809**	0.00	0.46	0.04	137.46	172.12	Enriched	Ala Ala Arg
**pos_871**	0.00	1.12	0.04	86.61	173.90	Enriched	4-Aminobutyraldehyde
**neg_1287**	0.01	0.97	0.04	218.43	391.67	Enriched	Deacetylcephalosporin C
**pos_1770**	0.09	0.63	0.04	3746.76	5321.92	Enriched	PA(16:0/0:0)[cyclic]
**pos_4746**	0.00	1.98	0.04	27.23	98.96	Enriched	Benzamide
**neg_6344**	0.01	4.61	0.04	23.37	584.64	Enriched	Baccatin III

**neg_6236**	0.01	1.03	0.04	294.69	532.46	Enriched	Leukotriene B4
**pos_989**	0.01	1.09	0.04	187.47	364.25	Enriched	Cytosine
**pos_8346**	0.00	0.88	0.04	180.46	314.01	Enriched	(6E,8E,12E,14E)-hexadeca-6,8,12,14-tetraen-10-ynoic acid
**pos_1674**	0.07	0.34	0.04	3194.73	3654.39	Enriched	all-trans-4-Hydroxyretinoic acid
**neg_6674**	0.04	1.06	0.04	1478.63	2786.31	Enriched	(±)9-HODE
**pos_5786**	0.00	2.05	0.04	7.11	25.72	Enriched	Ectocarpen
**pos_8738**	0.03	0.81	0.04	1143.16	1862.50	Enriched	3alpha,17alpha-Dihydroxy-5beta-pregnan-20-one
**pos_9183**	0.01	0.62	0.04	211.18	297.37	Enriched	p-cresol
**pos_7857**	0.00	0.54	0.04	72.52	96.34	Enriched	Hirsutine
**pos_1371**	0.00	4.22	0.04	0.92	17.88	Enriched	L-Pipecolate
**neg_6767**	0.03	1.61	0.04	771.83	2302.25	Enriched	Cucurbitacin D
**pos_5741**	0.00	1.22	0.04	21.57	45.15	Enriched	1,4-Dioxane-2,5-diol
**pos_9186**	0.00	0.65	0.04	120.95	173.46	Enriched	Limonene-1,2-diol
**neg_5985**	0.00	3.38	0.04	45.91	424.11	Enriched	Ankorine
**pos_8020**	0.01	0.48	0.04	287.65	371.11	Enriched	13-Octadecene-9,11-diynoic acid, (E)-; Exocarpic acid
**neg_3948**	0.00	1.85	0.04	60.47	196.80	Enriched	DL-Dithiothreitol
**pos_9504**	0.00	0.50	0.04	88.28	113.70	Enriched	2-Pentylfuran
**neg_6324**	0.01	3.59	0.04	43.57	532.76	Enriched	Adynerin
**pos_4476**	0.01	0.81	0.05	349.73	553.73	Enriched	Leukotriene E4
**pos_3690**	0.00	0.90	0.05	146.92	251.46	Enriched	Phe Ala
**pos_7670**	0.00	2.86	0.05	51.76	323.17	Enriched	Norwedelic acid
**pos_6118**	0.48	1.25	0.05	15052.18	33256.05	Enriched	5-(3E-Pentenyl)tetrahydro-2-oxo-3-furancarboxylic acid

**pos_7468**	0.00	2.70	0.05	16.97	107.10	Enriched	3beta-Hydroxyergosta-7,24(24(1))-dien-4alpha-carboxylate
**neg_4100**	0.05	0.97	0.05	1920.42	3469.62	Enriched	Fluorouracil
**pos_8739**	0.05	0.94	0.05	1931.83	3530.30	Enriched	CPA(18:2(9Z,12Z)/0:0)
**pos_3947**	0.00	1.17	0.05	52.79	108.81	Enriched	Abscisate
**pos_8649**	0.00	0.85	0.05	93.82	160.28	Enriched	Cannabigerolate
**pos_4264**	0.00	1.04	0.05	51.46	94.70	Enriched	L-Norleucine

Table S2B Differential analysis of metabolites in the rhizosphere soils between sexes.

otu_id	logCPM	logFC	PValue	MeanA	MeanB	level	
**neg_3511**	0.00	-10.52	0.00	248.70	0.00	Depleted	dTDP-3-methyl-4-oxo-2,6-dideoxy-L-allose
**pos_5990**	0.00	-3.80	0.00	56.34	4.34	Depleted	Violacein
**pos_4377**	0.00	-4.63	0.00	63.76	2.56	Depleted	Rhodomycin D
**pos_5918**	0.00	-5.46	0.00	7.79	0.00	Depleted	Ononin
**pos_6235**	0.00	-3.21	0.00	287.69	32.54	Depleted	Kanzonol D
**neg_7536**	0.00	-4.57	0.00	253.62	10.95	Depleted	Capsorubin
**pos_1066**	0.00	-4.24	0.00	219.28	11.60	Depleted	4-Guanidinobutyric acid
**pos_7178**	0.00	-9.10	0.00	96.68	0.00	Depleted	Nostoxanthin
**neg_3455**	0.00	-5.82	0.00	9.60	0.00	Depleted	5-Hydroxy-N-formylkynurenine
**pos_689**	0.00	-1.50	0.00	56.25	21.54	Depleted	Bergenin
**pos_9485**	0.52	-2.11	0.00	27924.59	6581.66	Depleted	Sporidesmolide I
**neg_6442**	0.00	-2.66	0.00	16.07	2.50	Depleted	3-Hydroxy-9,10-secoandrosta-1,3,5(10)-triene-9,17-dione
**pos_4373**	0.00	-4.38	0.00	3.62	0.00	Depleted	3-O-Methylisoetharine
**pos_4409**	0.01	-1.89	0.00	378.05	105.70	Depleted	4-Hydroxystyrene
**neg_4662**	0.00	-5.22	0.00	45.81	1.32	Depleted	7,8-Didemethyl-8-hydroxy-5-deazariboflavin
**pos_4415**	0.19	-1.87	0.00	9665.01	2734.89	Depleted	Phosphocreatine
**pos_3965**	0.00	-4.32	0.00	6.30	0.65	Depleted	Phenylalanyl-Glycine
**neg_3489**	0.01	-4.47	0.01	456.43	21.47	Depleted	PtdIns-(5)-P1 (1,2-dihexanoyl) (sodium salt)
**pos_9419**	0.04	-0.75	0.01	1735.21	1080.81	Depleted	3-Dimethylallyl-4-hydroxybenzaldehyde
**pos_3972**	0.00	-2.17	0.01	207.12	48.62	Depleted	N-Acetyl-L-2-amino-6-oxopimelate
**pos_4414**	0.26	-1.91	0.01	13598.83	3740.93	Depleted	3-Hydroxy-4-hydroxymethyl-2-methylpyridine-5-carboxylate
**neg_6389**	0.00	-4.71	0.01	4.74	0.00	Depleted	Crocin
**pos_552**	0.00	-2.14	0.01	15.45	3.61	Depleted	4-Aminobutanoate
**pos_7281**	0.00	-0.59	0.01	84.83	59.14	Depleted	Melperone
**neg_7347**	0.07	-5.66	0.01	4265.36	85.55	Depleted	[6]-Gingerol
**pos_4413**	0.07	-1.91	0.01	3729.44	1025.62	Depleted	3-Dehydroshikimate
**neg_3237**	0.00	-4.45	0.01	3.76	0.00	Depleted	FAD
**pos_3690**	0.00	-3.43	0.01	3.76	0.46	Depleted	Phe Ala
**neg_6866**	0.01	-4.80	0.01	739.16	28.29	Depleted	(4R)-7-Hydroxy-4-isopropenyl-7-methyl-2-oxo-oxepanone
**pos_5939**	0.00	-4.62	0.01	4.28	0.06	Depleted	N-Formimino-L-glutamate
**pos_6749**	0.00	-1.43	0.01	165.65	64.25	Depleted	Dhurrin
**neg_3906**	0.00	-3.91	0.01	2.61	0.00	Depleted	Valienone
**neg_6739**	0.00	-2.12	0.01	21.35	5.22	Depleted	Pennogenin

							3-O-beta-chacotrioside
**neg_1946**	0.00	-3.41	0.01	28.04	2.69	Depleted	Songorine
**pos_1743**	0.02	-1.14	0.01	953.37	452.86	Depleted	(3b,16a,21b,22a)-12-Oleanene-3,16,21,23,28-pentol-22-angeloyloxy-23-al
**pos_6669**	0.00	-3.54	0.02	14.64	1.49	Depleted	3-Methoxytyramine-betaxanthin
**pos_6525**	0.01	-1.88	0.02	511.85	141.10	Depleted	Blue pigment
**pos_4410**	0.05	-1.98	0.02	2584.57	676.12	Depleted	Isoquinoline
**pos_8356**	0.01	-0.37	0.02	536.69	436.42	Depleted	Santonine Oxime
**neg_4935**	0.00	-4.10	0.02	3.02	0.00	Depleted	Reduced riboflavin
**neg_6938**	0.00	-3.84	0.02	141.16	11.31	Depleted	19-Oxoandrost-4-ene-3,17-dione
**pos_7250**	0.02	-1.99	0.02	825.02	225.93	Depleted	Chenodeoxycholate
**neg_2857**	0.00	-3.74	0.02	2.19	0.00	Depleted	2-Furoate
**pos_5987**	0.00	-3.14	0.02	2.95	0.25	Depleted	Eriodictyol
**neg_3457**	0.24	-1.93	0.03	12227.58	3305.17	Depleted	C20913
**neg_3426**	0.00	-3.26	0.03	1.56	0.00	Depleted	Oxytetracycline
**neg_3304**	0.00	-3.25	0.03	1.53	0.00	Depleted	L-Arginine phosphate
**pos_481**	0.00	-1.94	0.03	13.42	3.58	Depleted	Fumarate
**pos_5848**	0.00	-3.79	0.03	2.26	0.00	Depleted	Glycitin
**pos_4678**	0.00	-3.43	0.03	3.48	0.41	Depleted	4'-O-Methylnorbelladine
**pos_7432**	0.00	-0.86	0.03	179.19	103.74	Depleted	Isotabtoxin
**pos_4738**	0.00	-3.56	0.03	2.14	0.00	Depleted	Pavetannin B2
**pos_7840**	0.00	-2.69	0.03	262.65	41.44	Depleted	Gravacridonol
**pos_6883**	0.06	-1.10	0.04	2779.42	1350.12	Depleted	Psilocin
**neg_4433**	0.00	-3.23	0.04	1.75	0.00	Depleted	Delphinidin 3-O-(6''-O-malonyl)-beta-glucoside-3'-O-beta-glucoside
**pos_1471**	0.00	-2.93	0.04	1.21	0.00	Depleted	N-octanoyl-L-Homoserine lactone
**pos_5368**	0.00	-2.54	0.04	3.46	0.71	Depleted	5-Hydroxykynurenamine
**pos_3974**	0.01	-2.24	0.04	434.99	97.13	Depleted	4-(3-Hydroxy-2-naphthyl)-2-oxobut-3-enoic acid
**neg_6415**	0.01	-2.72	0.04	528.92	85.42	Depleted	Haemanthamine
**pos_780**	0.00	-4.38	0.04	3.66	0.00	Depleted	UDP-3-O-(3-hydroxytetradecanoyl)-N-acetylglucosamine
**pos_5108**	0.00	-3.22	0.04	1.68	0.00	Depleted	Tetracenomycin D3
**neg_5266**	0.00	-2.84	0.04	43.76	6.86	Depleted	N-Acetylbialaphos
**pos_7221**	0.03	-0.93	0.04	1356.65	746.79	Depleted	Deoxyguanosine
**pos_4243**	0.00	-2.94	0.04	1.48	0.01	Depleted	Thr Ala Ala Arg
**pos_6936**	0.72	-1.25	0.04	32934.71	14368.04	Depleted	Cycluron
**pos_4281**	0.00	-2.29	0.05	8.10	1.73	Depleted	4'-Methoxy-2',3,7-trihydroxyisoflavanone
**pos_7946**	0.00	-2.90	0.05	189.81	27.00	Depleted	4-(3-Methyl-1-butenyl)-3,3',4',5-tetrahydroxystilbene

**pos_6527**	0.26	-0.96	0.05	11041.84	5930.03	Depleted	3-Acrylamidopropyl trimethylammonium
**neg_3962**	0.00	18.22	0.00	0.00	3.48	Enriched	2-Hydroxy-6-oxo-6-(2-hydroxyphenyl)-hexa-2,4-dienoate
**neg_910**	0.00	23.07	0.00	0.00	124.70	Enriched	PtdIns-(3,4)-P2 (1,2-dihexanoyl)
**pos_7157**	0.00	21.77	0.00	0.02	42.01	Enriched	Phenylacetylglycine
**neg_1298**	0.00	9.20	0.00	0.00	111.40	Enriched	dTDP-4-oxo-2-deoxy-beta-L-xylose
**pos_5106**	0.00	15.68	0.00	0.00	0.77	Enriched	5-Methylthiopentanaldoxime
**pos_7700**	0.22	4.57	0.00	547.98	14013.08	Enriched	Mucronine B
**pos_5667**	0.00	9.94	0.00	0.00	182.12	Enriched	Plaunotol
**pos_7697**	0.00	2.33	0.00	28.33	154.18	Enriched	Corticosterone
**pos_7692**	0.01	6.20	0.00	5.98	464.11	Enriched	Cyasterone
**neg_5649**	0.00	6.38	0.00	0.00	15.68	Enriched	(R)(-)-Allantoin
**pos_553**	0.01	3.42	0.00	54.65	644.45	Enriched	2-Aminomuconate semialdehyde
**pos_2795**	0.00	1.24	0.00	45.77	112.49	Enriched	Stigmastan-3,5-diene
**pos_6744**	0.00	5.25	0.00	3.12	127.35	Enriched	1-(5-Phospho-D-ribosyl)-5-amino-4-imidazolecarboxylate
**neg_845**	0.00	5.79	0.00	1.25	81.89	Enriched	3-Dehydro-D-glucose 6-phosphate
**pos_7698**	0.00	2.67	0.00	31.16	215.69	Enriched	Ajmalicine
**neg_5992**	0.00	6.90	0.00	0.00	23.87	Enriched	Phylloquinol
**pos_1319**	0.00	5.59	0.00	0.00	9.11	Enriched	DL-o-Tyrosine
**pos_8017**	0.00	4.28	0.00	13.83	282.44	Enriched	Daucosterol
**neg_7100**	0.00	2.48	0.00	26.91	164.79	Enriched	Alisol C monoacetate
**neg_1019**	0.00	4.30	0.00	2.29	47.50	Enriched	Aconitic acid
**pos_6942**	0.00	5.65	0.00	0.46	19.14	Enriched	Glycoursodeoxycholic acid
**pos_7680**	0.01	3.18	0.00	31.46	302.03	Enriched	Testosterone
**neg_6120**	0.00	4.67	0.00	0.00	5.17	Enriched	Lasalocid (sodium)
**pos_7695**	0.00	1.58	0.00	28.91	91.98	Enriched	5-Hydroxyindoleacetylglycine
**pos_4593**	0.00	3.22	0.00	14.55	147.15	Enriched	N-decanoyl-L-Homoserine lactone
**pos_980**	0.00	4.97	0.00	0.33	12.45	Enriched	QUINAMIDE ISOPROPYLIDENE
**pos_7408**	0.00	3.81	0.00	1.38	19.77	Enriched	20alpha,22beta-Dihydroxycholesterol
**pos_7242**	0.01	7.18	0.00	4.36	674.03	Enriched	5-O-beta-D-Mycaminosyltylactone
**pos_613**	0.07	1.44	0.00	1144.13	3345.27	Enriched	Betaine
**pos_7087**	0.00	5.84	0.00	0.00	10.56	Enriched	20,21-Diprenylterpendole I
**pos_3006**	0.27	0.97	0.00	5674.00	11991.02	Enriched	Ergosta-5,7,22,24(28)-tetraen-3beta-ol
**pos_740**	0.00	1.34	0.00	8.85	24.25	Enriched	L-Noradrenaline
**pos_6580**	0.00	5.85	0.00	0.00	11.71	Enriched	Neriifolin
**pos_3060**	0.02	1.42	0.00	322.93	898.26	Enriched	(22E,

							24x)-Ergosta-4,6,8,22-tetraen-3-one
**neg_6859**	0.00	3.34	0.00	18.34	211.58	Enriched	Demethyl-desacetyl-rifamycin S
**neg_7172**	0.00	2.86	0.00	17.13	137.92	Enriched	Streptomycin
**pos_6252**	0.00	2.70	0.00	1.53	11.28	Enriched	Ovalitenin B
**neg_6215**	0.00	5.90	0.00	0.00	12.43	Enriched	Gentamicin C1
**neg_3271**	0.00	4.37	0.00	0.00	4.05	Enriched	Methyl jasmonate
**neg_6804**	0.00	5.16	0.01	0.00	6.60	Enriched	Bergamottin
**neg_5122**	0.00	3.80	0.01	0.00	2.81	Enriched	Genipin 1-????-D-gentiobioside
**pos_8388**	0.00	8.83	0.01	0.00	89.07	Enriched	ADP
**pos_7623**	0.00	1.40	0.01	61.23	170.36	Enriched	Phylloquinone
**pos_7938**	0.01	3.94	0.01	28.66	479.00	Enriched	(1R,10aS)-1,4,10,10a-Tetrahydrophenazine-1-carboxylate
**neg_1230**	0.00	3.96	0.01	0.75	11.29	Enriched	D-Glucuronolactone
**neg_6868**	0.05	1.91	0.01	623.75	2614.26	Enriched	1-Oleoyl-sn-glycero-3-phosphocholine
**pos_7272**	0.00	7.86	0.01	0.50	89.89	Enriched	Avermectin A1a
**pos_8383**	0.00	8.13	0.01	0.00	54.14	Enriched	Ethanolamine phosphate
**pos_992**	0.00	1.85	0.01	4.07	15.25	Enriched	2-Deoxy-scyllo-inosamine
**neg_6146**	0.00	4.13	0.01	0.00	3.36	Enriched	19-Oxotestosterone
**neg_2184**	0.00	4.38	0.01	0.48	7.39	Enriched	Antheraxanthin
**pos_1311**	0.00	1.97	0.01	5.38	22.42	Enriched	N-(5-Methyl-3-oxohexyl)alanine
**pos_9283**	0.00	4.82	0.01	0.00	5.17	Enriched	2-C-Methyl-D-erythritol 4-phosphate
**pos_590**	0.00	1.40	0.01	29.08	81.16	Enriched	L-Carnitine
**pos_7362**	0.00	3.38	0.01	1.04	11.15	Enriched	Calcitriol
**neg_4629**	0.00	3.62	0.01	0.00	2.20	Enriched	8-?O-?Acetylharpagide
**neg_6199**	0.01	1.22	0.01	231.45	575.46	Enriched	3alpha,7alpha,12alpha-Trihydroxy-5beta-cholestanoate
**neg_7235**	0.00	2.04	0.01	37.49	166.19	Enriched	Neomycin B
**pos_8387**	0.00	4.79	0.01	6.25	176.70	Enriched	N3-Fumaroyl-L-2,3-diaminopropanoate
**neg_5036**	0.00	3.59	0.01	0.00	2.19	Enriched	Pentalenolactone
**neg_3284**	0.00	4.13	0.01	0.00	3.44	Enriched	Pelargonidin 3-O-(6-O-malonyl-beta-D-glucoside)
**neg_5550**	0.00	4.42	0.02	0.00	3.85	Enriched	Pelargonidin 3-O-glucoside
**pos_860**	0.01	1.42	0.02	112.47	330.68	Enriched	Benz[c]acridine
**neg_1215**	0.00	1.64	0.02	5.99	19.43	Enriched	Malonylglycitin
**pos_7947**	0.78	2.96	0.02	5424.45	45807.71	Enriched	Khellol glucoside
**neg_6292**	0.07	1.83	0.02	958.70	3775.12	Enriched	Salmeterol
**pos_7943**	0.20	2.89	0.02	1460.28	11703.91	Enriched	Leflunomide
**pos_5888**	0.00	3.37	0.02	0.15	1.83	Enriched	(1'S,5'S)-5'-Hydroxyaverantin

**pos_7451**	0.00	3.54	0.02	24.24	286.90	Enriched	2'-N-Acetylparomamine
**neg_4971**	0.00	3.39	0.02	0.00	2.16	Enriched	Curcumin
**neg_1064**	0.00	3.10	0.02	0.00	1.60	Enriched	Quercetin 3-O-(6-O-malonyl-beta-D-glucoside)
**pos_1188**	0.00	3.88	0.02	0.27	5.43	Enriched	Terbutaline-1-sulfate
**pos_5213**	0.00	2.82	0.02	0.63	5.01	Enriched	His-Tyr-OH
**neg_1408**	0.00	2.32	0.02	6.71	36.36	Enriched	7,8-Dihydroxanthopterin
**pos_7349**	0.00	0.96	0.02	79.58	165.08	Enriched	((2S,3S,4R,7S,10S)-4-ethyl-3,7,10-trimethyldodecahydrocyclopenta[b]quinolizin-2-yl)methanol
**pos_6606**	0.00	3.11	0.03	0.00	1.79	Enriched	28-Homobrassinolide
**neg_1921**	0.00	2.97	0.03	13.88	120.31	Enriched	7alpha,12alpha-Dihydroxy-5beta-cholestan-3-one
**neg_6582**	0.00	4.65	0.03	0.00	4.77	Enriched	Germacrene A acid
**neg_6771**	0.02	1.43	0.03	382.43	1092.10	Enriched	Traumatin
**pos_2786**	0.01	0.65	0.03	144.25	235.65	Enriched	Gypenoside XVII
**neg_4136**	0.00	4.40	0.03	0.00	4.49	Enriched	Nebramycin factor 4
**pos_8306**	0.06	1.16	0.03	1122.54	2742.10	Enriched	PC(18:1(9E)/0:0)[U]
**neg_4021**	0.00	1.79	0.03	1.72	5.76	Enriched	3,6,8-Trimethylallantoin
**neg_6187**	0.00	3.63	0.03	0.00	2.26	Enriched	Bryostatin 1
**neg_1274**	0.00	3.81	0.03	0.00	2.77	Enriched	5-Phosphooxy-L-lysine
**pos_5017**	0.01	0.46	0.03	327.56	476.26	Enriched	Elenaic acid
**pos_6897**	0.00	2.80	0.03	5.59	40.09	Enriched	Prolyl-2-naphthylamide
**pos_1539**	0.00	2.31	0.03	5.84	29.08	Enriched	7-Ethyl-2,3,4,5,6,7-hexahydrocyclopent[b]azepin-8(1H)-one
**neg_7311**	0.00	3.56	0.03	4.66	58.41	Enriched	Diosgenin glucoside
**pos_8093**	0.00	3.32	0.03	0.00	1.94	Enriched	7-Dehydrodesmosterol
**neg_7249**	0.00	1.80	0.03	11.01	41.26	Enriched	27-O-Demethyl-25-O-desacetylrifamycin SV
**neg_6578**	0.00	1.50	0.04	16.11	50.52	Enriched	Brassinolide
**pos_7675**	0.00	3.25	0.04	0.80	10.81	Enriched	PE(P-16:0/0:0)
**pos_1340**	0.00	3.39	0.04	0.07	2.00	Enriched	Isopentenyladenine-9-N-glucoside
**neg_7273**	0.01	2.05	0.04	162.42	706.16	Enriched	Nebramycin 5'
**pos_1881**	0.00	3.07	0.04	0.00	1.59	Enriched	Clavaminic acid
**neg_7122**	0.06	0.97	0.04	1218.44	2537.41	Enriched	2,2'-Diketospirilloxanthin
**neg_4565**	0.00	3.12	0.04	0.00	1.67	Enriched	Rutaevin
**pos_9124**	0.01	2.29	0.04	153.15	790.91	Enriched	Sipeimine-3beta-D-glucoside
**pos_8543**	0.00	0.82	0.04	86.71	164.72	Enriched	D-Urobilinogen
**pos_6219**	0.00	1.91	0.05	2.64	10.57	Enriched	7-Hydroxy-6-methyl-8-ribityllumazine
**pos_2819**	0.00	1.01	0.05	105.51	223.35	Enriched	14-Demethyllanosterol
**neg_3603**	0.00	2.79	0.05	0.00	1.21	Enriched	Cyanidin

							3-O-(6-O-p-coumaroyl)glucoside
**neg_5947**	0.01	3.81	0.05	41.71	580.54	Enriched	Dethiobiotin
**pos_1321**	0.00	1.28	0.05	9.73	24.94	Enriched	2-Hydroxycinnamic acid

Table S3A Bacterial 16S rRNA Sequencing Data Quality Assessment.

Sample ID	Raw Reads	Clean Reads	Effective Reads	AvgLen(bp)	GC(%)	Q20(%)	Q30(%)	Effective(%)	OTU_Num	Coverage
FR01	79866	79577	65999	402	54.66	99.31	96.99	82.64	1612	0.9985
FR02	79849	79546	69421	402	54.86	99.31	97	86.94	852	0.9987
FR03	80242	79978	67377	401	53.96	99.31	96.96	83.97	1767	0.9983
FRh01	79494	79196	76161	416	57.22	99.12	96.38	95.81	824	0.9975
FRh02	80040	79727	77684	417	57.21	99.12	96.37	97.06	894	0.9979
FRh03	80280	80026	78390	418	57.3	99.11	96.34	97.65	1521	0.998
MR01	80189	79917	65546	401	54.42	99.33	97.02	81.74	844	0.9987
MR02	80319	80010	65421	402	54.25	99.33	97.04	81.45	1700	0.9981
MR03	80018	79699	60171	402	53.85	99.31	96.95	75.2	1690	0.9976
MRh01	79598	79317	75799	417	56.15	99.12	96.34	95.23	879	0.9979
MRh02	79900	79581	76706	416	55.64	99.11	96.34	96	1662	0.9979
MRh03	80167	79841	77543	417	56.19	99.1	96.28	96.73	869	0.9976

Table S3B. Fungi ITS1 Sequencing Data Quality Assessment.

Sample ID	Raw Reads	Clean Reads	Effective Reads	AvgLen(bp)	GC(%)	Q20(%)	Q30(%)	Effective(%)	OTU_Num	Coverage
FR01	54094	53834	53604	299	40.85	99.74	98.34	99.09	294	0.9995
FR02	51183	50930	50705	231	47.48	99.96	99.67	99.07	320	0.999
FR03	62096	61796	61626	278	48.87	99.68	98.42	99.24	303	0.9997
FRh01	27594	27432	27080	243	45.29	99.85	99.12	98.14	503	0.9967
FRh02	33624	33414	33069	249	45.82	99.84	99.06	98.35	557	0.9991
FRh03	26284	26121	25765	236	46.09	99.89	99.32	98.03	420	0.9976
MR01	69543	69125	68487	261	42.95	99.9	99.29	98.48	291	0.9991
MR02	48795	48575	48131	233	49.57	99.92	99.52	98.64	268	0.999
MR03	37503	37310	37086	253	44.35	99.9	99.3	98.89	270	0.9988
MRh01	27518	27366	26999	236	46.49	99.89	99.31	98.11	599	0.9962
MRh02	44845	44448	43983	252	45.37	99.85	99.07	98.08	638	0.9993
MRh03	28306	28143	27796	239	46.01	99.88	99.27	98.2	619	0.9969
Sum/ave	511385	508494	504331	251	45.76	99.86	99.14	98.53	1021	0.9984

Table S4. Comparison of relative abundance of bacteria and fungi in each group.

	phyla	FR	FRh	MR	MRh
Bacteria	Proteobacteria	**49.9 a**	11.1 b	**55.1 a**	10.8 b
Bacteria	Acidobacteriota	12.6 b	**39.3 a**	4.4 b	**31.9 a**
Bacteria	Actinobacteriota	17.4 a	14.7 a	28.2 a	14.8 a
Bacteria	Firmicutes	2.6 a	4.4 a	1.2 a	17.6 a
Bacteria	Gemmatimonadota	3.8 a	6.9 a	1.8 a	6.2 a
Bacteria	Verrucomicrobiota	0 a	4.7 a	0 a	5.4 a
Bacteria	Myxococcota	**7 a**	0.8 bc	4.5 ab	0.4 c
Bacteria	Chloroflexi	1.4 ab	4.7 a	0.4 b	4.3 ab
Bacteria	Patescibacteria	1.2 a	0.5 a	0.9 a	1.4 a
Bacteria	Others	4.2 b	12.9 a	3.4 b	7.3 ab
Fungi	Aphelidiomycota	0 a	0 a	0 a	0 a
Fungi	Ascomycota	59.5 a	66.3 a	66.7 a	66 a
Fungi	Basidiomycota	33.3 a	17.1 a	30.8 a	15.2 a
Fungi	Chytridiomycota	0.5 b	**3.5 a**	0.2 b	**2.6 a**
Fungi	Glomeromycota	0.1 ab	0.2 a	0 b	0.2 ab
Fungi	Kickxellomycota	0.1 a	0.1 a	0.2 a	0.2 a
Fungi	Mortierellomycota	1 a	5 a	0.5 a	9.8 a
Fungi	Mucoromycota	0 a	0.1 a	0 a	0.1 a
Fungi	Olpidiomycota	0 a	0 a	0 a	0.1 a
Fungi	Others	5.6 a	7.7 a	1.6 a	5.9 a

Table S5A Differential analysis of bacteria in roots between sexes.

otu_id	baseMean	log2FC	lfcSE	stat	pvalue	padj	
**unclassified_Xanthomonadaceae**	96.53	-6.60	2.01	-3.29	0.00	0.06	Depleted
**unclassified_Gammaproteobacteria**	7.32	-3.00	1.10	-2.72	0.01	0.16	Depleted
**Candidatus_Solibacter**	848.59	-1.22	0.54	-2.24	0.02	0.26	Depleted
**Dactylosporangium**	74.93	-2.44	1.09	-2.23	0.03	0.26	Depleted
**Escherichia_Shigella**	29.84	-1.33	0.60	-2.21	0.03	0.26	Depleted
**Parabacteroides**	6.86	-2.59	1.17	-2.22	0.03	0.26	Depleted
**Rodentibacter**	52.84	-1.87	0.83	-2.25	0.02	0.26	Depleted
**unclassified_A4b**	47.32	-2.32	1.04	-2.23	0.03	0.26	Depleted
**unclassified_Burkholderiales**	4.61	-5.77	2.55	-2.27	0.02	0.26	Depleted
**uncultured_proteobacterium**	16.15	-2.78	1.22	-2.28	0.02	0.26	Depleted
**Polycyclovorans**	3.53	-3.68	1.77	-2.07	0.04	0.32	Depleted
**unclassified_Myxococcales**	3.82	-4.21	2.12	-1.98	0.05	0.36	Depleted
**Mitsuaria**	261.05	4.30	0.95	4.50	0.00	0.00	Enriched
**Streptomyces**	2063.24	3.03	0.77	3.95	0.00	0.01	Enriched
**Brevundimonas**	20.15	4.12	1.27	3.24	0.00	0.06	Enriched
**Methylophilus**	47.67	4.95	1.50	3.29	0.00	0.06	Enriched
**Shimazuella**	9.27	4.31	1.33	3.25	0.00	0.06	Enriched
**Chryseobacterium**	424.98	4.65	1.48	3.15	0.00	0.07	Enriched
**Allorhizobium_Neorhizobium_Pararhizobium_Rhizobium**	2241.32	2.46	0.80	3.06	0.00	0.07	Enriched
**Mycobacterium**	371.19	1.38	0.45	3.06	0.00	0.07	Enriched
**Agromyces**	6.24	4.74	1.68	2.82	0.00	0.15	Enriched
**Actinocorallia**	9.42	4.77	1.79	2.67	0.01	0.16	Enriched
**Hirschia**	38.45	5.20	1.97	2.63	0.01	0.16	Enriched
**Roseomonas**	19.02	3.51	1.31	2.69	0.01	0.16	Enriched
**Sandaracinus**	14.24	2.86	1.06	2.70	0.01	0.16	Enriched
**Uliginosibacterium**	12.47	2.57	0.98	2.63	0.01	0.16	Enriched
**Dyadobacter**	34.74	2.51	0.99	2.54	0.01	0.20	Enriched
**Niastella**	1558.09	2.44	0.97	2.51	0.01	0.20	Enriched
**Micromonospora**	28.06	2.61	1.06	2.47	0.01	0.22	Enriched
**Edaphobaculum**	18.48	3.34	1.51	2.21	0.03	0.26	Enriched
**Microvirga**	7.72	2.73	1.23	2.23	0.03	0.26	Enriched
**Pseudonocardia**	37.26	1.44	0.65	2.23	0.03	0.26	Enriched
**unclassified_Acidobacterium_sp._WY65**	8.06	2.92	1.24	2.35	0.02	0.26	Enriched
**unclassified_env.OPS_17**	96.93	3.15	1.42	2.22	0.03	0.26	Enriched
**AAP99**	118.84	2.22	1.02	2.17	0.03	0.27	Enriched
**Virgisporangium**	13.78	3.15	1.46	2.17	0.03	0.27	Enriched
**unclassified_Hyphomicrobiaceae**	3.91	4.18	1.96	2.13	0.03	0.29	Enriched
**unclassified_Methylophilaceae**	42.29	1.88	0.91	2.06	0.04	0.32	Enriched
**Massilia**	167.79	2.27	1.12	2.02	0.04	0.35	Enriched

**uncultured_Caldilineaceae_bacterium**	3.57	4.11	2.06	2.00	0.05	0.36	Enriched

Table S5B Differential analysis of bacteria in rhizosphere soils between sexes.

otu_id	ıseMean	log2FC	lfcSE	stat	pvalue	padj	
**unclassified_KF_JG30_B3**	108.70	-1.74	0.45	-3.84	0.00	0.01	Depleted
**uncultured_Holophaga_sp.**	157.22	-2.19	0.57	-3.82	0.00	0.01	Depleted
**P3OB_42**	86.78	-1.84	0.49	-3.76	0.00	0.01	Depleted
**unclassified_Elsterales**	665.66	-1.65	0.46	-3.60	0.00	0.01	Depleted
**unclassified_Ktedonobacteraceae**	75.52	-3.93	1.25	-3.15	0.00	0.03	Depleted
**uncultured_proteobacterium**	361.20	-2.02	0.64	-3.14	0.00	0.03	Depleted
**unclassified_Bacteria**	2877.17	-1.20	0.39	-3.10	0.00	0.03	Depleted
**unclassified_Armatimonadales**	4.51	-6.28	2.05	-3.06	0.00	0.03	Depleted
**unclassified_Alphaproteobacteria**	326.82	-1.26	0.42	-3.01	0.00	0.03	Depleted
**uncultured_gamma_proteobacterium**	69.33	-1.41	0.48	-2.96	0.00	0.04	Depleted
**unclassified_A21b**	235.10	-1.89	0.65	-2.89	0.00	0.04	Depleted
**Haliangium**	1018.48	-1.20	0.42	-2.88	0.00	0.04	Depleted
**unclassified_Rokubacteriales**	71.48	-1.67	0.59	-2.86	0.00	0.04	Depleted
**ADurb.Bin063_1**	329.82	-1.39	0.49	-2.82	0.00	0.05	Depleted
**unclassified_Isosphaeraceae**	100.42	-2.00	0.72	-2.80	0.01	0.05	Depleted
**unclassified_Subgroup_2**	194.55	-1.82	0.65	-2.78	0.01	0.05	Depleted
**Candidatus_Solibacter**	1932.80	-0.94	0.35	-2.68	0.01	0.06	Depleted
**Tumebacillus**	78.19	-2.23	0.83	-2.69	0.01	0.06	Depleted
**uncultured_Desulfovirga_sp.**	395.73	-2.00	0.74	-2.69	0.01	0.06	Depleted
**Ellin516**	117.57	-1.31	0.49	-2.66	0.01	0.06	Depleted
**MND1**	470.05	-1.07	0.41	-2.60	0.01	0.07	Depleted
**Roseiarcus**	116.43	-1.90	0.73	-2.60	0.01	0.07	Depleted
**uncultured_actinobacterium**	235.20	-2.04	0.80	-2.57	0.01	0.07	Depleted
**unclassified_Subgroup_17**	55.16	-2.00	0.81	-2.46	0.01	0.08	Depleted
**Sumerlaea**	143.68	-1.77	0.74	-2.40	0.02	0.09	Depleted
**uncultured_forest_soil_bacterium**	581.51	-1.72	0.72	-2.39	0.02	0.09	Depleted
**unclassified_Vicinamibacterales**	2428.79	-0.88	0.37	-2.39	0.02	0.09	Depleted
**unclassified_Acidobacteriales**	3937.21	-0.84	0.36	-2.37	0.02	0.10	Depleted
**unclassified_JG30_KF_AS9**	443.19	-1.22	0.52	-2.36	0.02	0.10	Depleted
**uncultured_Firmicutes_bacterium**	83.97	-1.85	0.79	-2.33	0.02	0.10	Depleted
**uncultured_Ferrimicrobium_sp.**	79.09	-1.24	0.53	-2.33	0.02	0.10	Depleted
**Anaeromyxobacter**	52.83	-1.58	0.69	-2.29	0.02	0.11	Depleted
**unclassified_Gemmataceae**	155.37	-0.88	0.39	-2.27	0.02	0.11	Depleted
**unclassified_Xanthobacteraceae**	1189.36	-0.72	0.32	-2.26	0.02	0.11	Depleted
**unclassified_Gemmatimonadaceae**	1362.16	-1.07	0.47	-2.25	0.02	0.12	Depleted
**unclassified_Methyloligellaceae**	81.59	-0.90	0.41	-2.19	0.03	0.13	Depleted
**unclassified_SC_I_84**	1825.06	-0.83	0.38	-2.18	0.03	0.13	Depleted
**unclassified_Subgroup_13**	75.94	-2.46	1.13	-2.17	0.03	0.13	Depleted
**unclassified_Simkaniaceae**	34.40	-1.89	0.89	-2.12	0.03	0.14	Depleted
**SM1A02**	59.42	-1.08	0.52	-2.09	0.04	0.15	Depleted

**Nitrospira**	539.27	-0.78	0.38	-2.02	0.04	0.17	Depleted
**Rhodoplanes**	241.27	-0.94	0.47	-2.02	0.04	0.17	Depleted
**uncultured_Alphaproteobacteria_bacterium**	26.71	-1.85	0.92	-2.02	0.04	0.17	Depleted
**uncultured_Solibacterales_bacterium**	11.15	-1.95	0.97	-2.01	0.04	0.17	Depleted
**unclassified_IMCC26256**	306.09	-0.95	0.48	-1.97	0.05	0.18	Depleted
**Allorhizobium_Neorhizobium_Pararhizobium_Rhizobium**	227.47	2.67	0.57	4.68	0.00	0.00	Enriched
**Flavitalea**	23.75	4.97	1.03	4.83	0.00	0.00	Enriched
**Ramlibacter**	211.47	2.23	0.47	4.70	0.00	0.00	Enriched
**Variovorax**	70.38	2.61	0.57	4.56	0.00	0.00	Enriched
**Paenibacillus**	27.25	3.02	0.71	4.26	0.00	0.00	Enriched
**Flavisolibacter**	312.68	1.91	0.45	4.22	0.00	0.00	Enriched
**Caulobacter**	24.03	2.98	0.75	3.99	0.00	0.00	Enriched
**Devosia**	88.28	1.63	0.44	3.68	0.00	0.01	Enriched
**Luteimonas**	15.75	4.89	1.32	3.70	0.00	0.01	Enriched
**Dyadobacter**	36.55	4.29	1.18	3.63	0.00	0.01	Enriched
**Lysobacter**	121.92	1.99	0.55	3.60	0.00	0.01	Enriched
**Bosea**	27.65	3.42	0.97	3.52	0.00	0.01	Enriched
**unclassified_Rhodanobacteraceae**	18.31	4.51	1.28	3.53	0.00	0.01	Enriched
**Hirschia**	11.48	5.13	1.50	3.43	0.00	0.01	Enriched
**unclassified_Intrasporangiaceae**	233.73	1.32	0.39	3.36	0.00	0.02	Enriched
**Aridibacter**	162.06	2.26	0.68	3.30	0.00	0.02	Enriched
**Luteitalea**	11.78	5.33	1.62	3.30	0.00	0.02	Enriched
**Pseudorhodoferax**	9.50	4.97	1.52	3.27	0.00	0.02	Enriched
**Rhodocytophaga**	6.18	5.97	1.85	3.23	0.00	0.02	Enriched
**Bacteroides**	47.60	2.86	0.90	3.20	0.00	0.02	Enriched
**Segetibacter**	18.21	2.95	0.93	3.19	0.00	0.02	Enriched
**Olivibacter**	14.07	3.86	1.25	3.10	0.00	0.03	Enriched
**unclassified_Sandaracinaceae**	42.08	2.81	0.92	3.05	0.00	0.03	Enriched
**unclassified_Oxalobacteraceae**	36.82	2.68	0.88	3.04	0.00	0.03	Enriched
**Aliihoeflea**	11.74	2.96	0.98	3.01	0.00	0.03	Enriched
**Cupriavidus**	275.12	1.57	0.53	2.96	0.00	0.04	Enriched
**Bifidobacterium**	27.89	3.82	1.30	2.94	0.00	0.04	Enriched
**unclassified_Microscillaceae**	18.85	3.36	1.16	2.89	0.00	0.04	Enriched
**Achromobacter**	19.66	3.85	1.35	2.86	0.00	0.04	Enriched
**Faecalibacterium**	26.24	3.04	1.07	2.84	0.00	0.05	Enriched
**Filimonas**	16.21	2.21	0.78	2.83	0.00	0.05	Enriched
**unclassified_Oscillospiraceae**	20.24	2.99	1.06	2.81	0.00	0.05	Enriched
**Lactobacillus**	79.85	1.58	0.57	2.79	0.01	0.05	Enriched
**Muribaculum**	6.08	5.53	2.00	2.76	0.01	0.05	Enriched
**Nocardioides**	165.91	1.05	0.39	2.71	0.01	0.06	Enriched
**uncultured_Bacteroidales_bacterium**	34.11	2.34	0.86	2.72	0.01	0.06	Enriched

**Ohtaekwangia**	7.86	5.87	2.20	2.67	0.01	0.06	Enriched
**unclassified_Micrococcaceae**	163.48	1.19	0.45	2.67	0.01	0.06	Enriched
**Chryseolinea**	4.27	5.44	2.06	2.65	0.01	0.06	Enriched
**Aeromicrobium**	22.24	1.99	0.77	2.57	0.01	0.07	Enriched
**JGI_0001001_H03**	155.30	1.85	0.72	2.58	0.01	0.07	Enriched
**Sphingobium**	12.83	2.60	1.00	2.59	0.01	0.07	Enriched
**unclassified_Sphingobacteriaceae**	4.71	6.23	2.42	2.58	0.01	0.07	Enriched
**unclassified_env.OPS_17**	7.78	5.11	1.99	2.57	0.01	0.07	Enriched
**Paracoccus**	15.86	3.72	1.45	2.56	0.01	0.07	Enriched
**Bacillus**	1814.90	2.08	0.82	2.53	0.01	0.07	Enriched
**unclassified_Enterobacteriaceae**	79.01	1.75	0.71	2.46	0.01	0.08	Enriched
**Colidextribacter**	16.67	3.36	1.39	2.42	0.02	0.09	Enriched
**Microvirga**	14.78	2.48	1.03	2.41	0.02	0.09	Enriched
**unclassified_Saprospiraceae**	4.03	3.35	1.39	2.40	0.02	0.09	Enriched
**unclassified_mle1_27**	5.36	4.47	1.85	2.41	0.02	0.09	Enriched
**Novosphingobium**	48.49	1.41	0.60	2.36	0.02	0.10	Enriched
**unclassified_Lachnospiraceae**	13.33	1.98	0.84	2.36	0.02	0.10	Enriched
**Rummeliibacillus**	5.92	4.49	1.91	2.35	0.02	0.10	Enriched
**Arenimonas**	11.41	2.28	0.98	2.33	0.02	0.10	Enriched
**Niabella**	5.39	3.57	1.56	2.28	0.02	0.11	Enriched
**Stenotrophobacter**	7.81	2.85	1.28	2.22	0.03	0.12	Enriched
**YC_ZSS_LKJ147**	61.96	2.67	1.20	2.23	0.03	0.12	Enriched
**Pseudoxanthomonas**	17.69	2.45	1.11	2.21	0.03	0.13	Enriched
**Brevundimonas**	37.85	2.53	1.17	2.17	0.03	0.13	Enriched
**unclassified_Burkholderiales**	14.53	2.37	1.09	2.17	0.03	0.13	Enriched
**Prevotellaceae_UCG_001**	13.28	2.04	0.94	2.16	0.03	0.13	Enriched
**unclassified_A4b**	146.38	2.21	1.03	2.15	0.03	0.13	Enriched
**Pseudofulvimonas**	4.28	5.99	2.80	2.14	0.03	0.14	Enriched
**unclassified_Caldilineaceae**	9.63	2.04	0.96	2.13	0.03	0.14	Enriched
**unclassified_Vampirovibrionaceae**	5.36	3.69	1.73	2.14	0.03	0.14	Enriched
**unclassified_Rhizobiaceae**	46.83	1.72	0.83	2.08	0.04	0.15	Enriched
**Rothia**	6.69	5.30	2.55	2.08	0.04	0.15	Enriched
**Thermomonas**	4.72	4.23	2.06	2.05	0.04	0.16	Enriched
**unclassified_Blastocatellaceae**	411.39	1.38	0.67	2.04	0.04	0.16	Enriched
**Chitinophaga**	108.16	1.47	0.74	2.00	0.05	0.17	Enriched
**Lachnospiraceae_NK4A136_group**	31.87	1.41	0.71	2.00	0.05	0.17	Enriched
**uncultured_Bacteroidetes_bacterium**	4.44	3.08	1.56	1.97	0.05	0.18	Enriched
**unclassified_Muribaculaceae**	98.85	1.20	0.61	1.96	0.05	0.18	Enriched

Table S5C. Differential analysis of fungi in roots between sexes.

otu_id	baseMean	log2FC	lfcSE	stat	pvalue	padj	
**unclassified_Basidiomycota**	543.89	-3.87	0.83	-4.65	0.00	0.00	Depleted
**unclassified_Sordariomycetes**	377.32	-3.60	1.09	-3.31	0.00	0.02	Depleted
**Candida**	56.87	-2.05	0.66	-3.11	0.00	0.03	Depleted
**Solicoccozyma**	34.39	-3.21	1.05	-3.05	0.00	0.03	Depleted
**Trechispora**	64.98	-6.40	2.14	-2.99	0.00	0.04	Depleted
**Rhodotorula**	36.04	-2.39	0.82	-2.90	0.00	0.05	Depleted
**unclassified_Chytridiomycota**	32.20	-4.38	1.53	-2.87	0.00	0.05	Depleted
**unclassified_Mortierellaceae**	13.92	-4.51	1.69	-2.67	0.01	0.08	Depleted
**Malassezia**	21.45	-4.90	1.87	-2.62	0.01	0.08	Depleted
**Sporidiobolus**	7.97	-5.36	2.19	-2.45	0.01	0.11	Depleted
**Acremonium**	494.92	-1.77	0.84	-2.11	0.03	0.23	Depleted
**unclassified_Auriculariales**	10.13	-4.57	2.17	-2.11	0.03	0.23	Depleted
**Filobasidium**	95.70	-1.91	0.91	-2.09	0.04	0.23	Depleted
**Ilyonectria**	25.72	-1.96	0.99	-1.99	0.05	0.26	Depleted
**Conocybe**	25.30	-2.21	1.12	-1.96	0.05	0.27	Depleted
**unclassified_Ceratobasidiaceae**	9076.97	9.65	1.46	6.62	0.00	0.00	Enriched
**unclassified_Plectosphaerellaceae**	151.83	4.26	1.02	4.18	0.00	0.00	Enriched
**Cladosporium**	4199.83	2.88	0.77	3.73	0.00	0.01	Enriched
**unclassified_Fungi**	978.09	2.07	0.58	3.56	0.00	0.01	Enriched
**Paraconiothyrium**	26.62	5.42	1.72	3.15	0.00	0.03	Enriched
**unclassified_Ascomycota**	1689.24	3.71	1.20	3.10	0.00	0.03	Enriched
**Xenomyrothecium**	89.07	5.18	1.87	2.76	0.01	0.06	Enriched
**Gibellulopsis**	1063.37	2.93	1.11	2.64	0.01	0.08	Enriched
**Wallemia**	274.26	4.51	1.78	2.53	0.01	0.10	Enriched
**Curvularia**	45.17	2.08	0.88	2.36	0.02	0.14	Enriched
**Colletotrichum**	13.50	4.20	1.91	2.19	0.03	0.20	Enriched
**Neomicrosphaeropsis**	6.58	4.64	2.25	2.06	0.04	0.24	Enriched
**Vishniacozyma**	45.32	3.01	1.47	2.04	0.04	0.24	Enriched

Table S5D. Differential analysis of fungi in rhizosphere soils between sexes.

otu_id	baseMean	log2FoldChange	lfcSE	stat	pvalue	padj	
**Cymostachys**	40.34133543	19.39697989	3.226643235	6.011504365	1.84E-09	5.40E-07	Enriched
**Exserohilum**	161.9406826	9.655372301	1.780498753	5.42284699	5.87E-08	8.62E-06	Enriched
**Acrocalymma**	249.8620315	14.99039183	3.235066476	4.633719877	3.59E-06	0.00035197	Enriched
**Cortinarius**	17.3373386	7.098561819	1.942158786	3.654985303	0.000257197	0.018903958	Enriched
**Sampaiozyma**	7.325896093	6.342913496	1.963570769	3.230295335	0.001236624	0.072713495	Enriched
**Inocybe**	44.22730949	5.061234659	1.87741042	2.69585947	0.007020728	0.344015668	Enriched
**Olpidium**	4.982613145	4.028810706	1.745043997	2.308715833	0.020959355	0.768209918	Enriched
**Pseudaleuria**	16.63474315	3.19929882	1.412534106	2.264935626	0.02351663	0.768209918	Enriched
**unclassified_Auriculariales**	10.12997938	4.957998009	2.138867253	2.318048492	0.020446686	0.768209918	Enriched

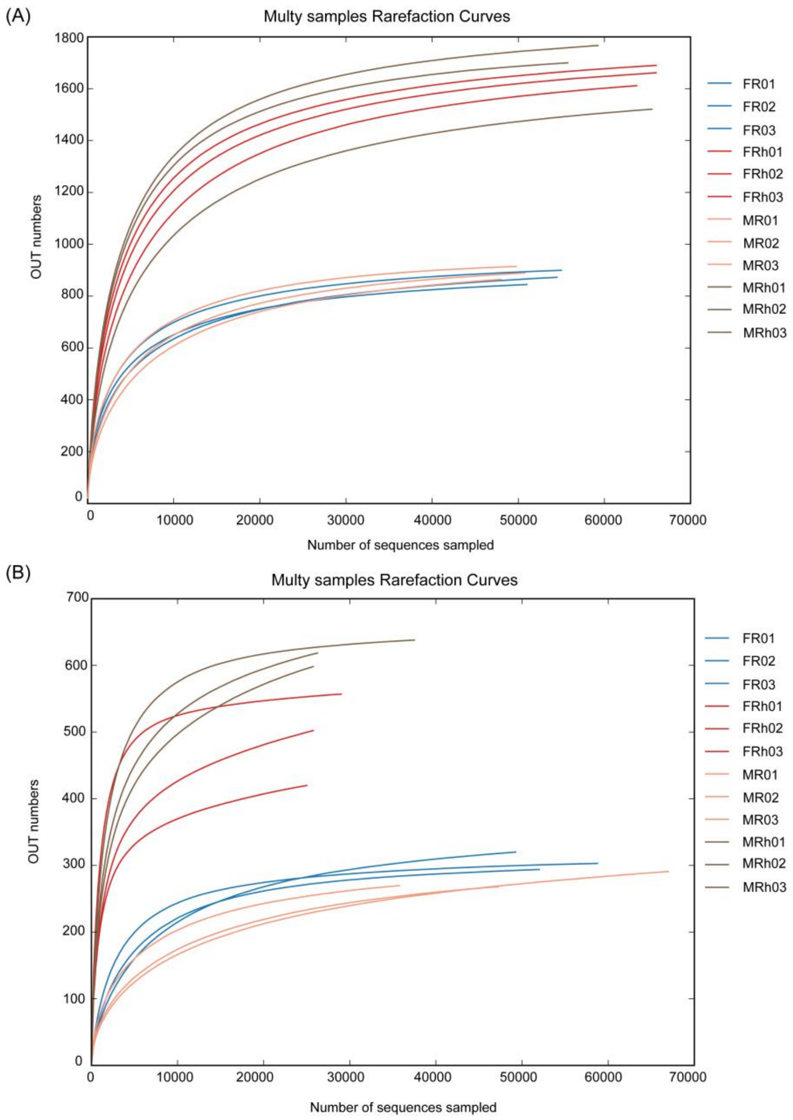

Figure S1 Rarefaction curves for bacterial (A) and fungal (B) OTUs.
